# Divergent encoding of active avoidance behavior in corticostriatal and corticolimbic projections

**DOI:** 10.1038/s41598-022-14930-3

**Published:** 2022-06-24

**Authors:** Bridget L. Kajs, Adrienne C. Loewke, Jeffrey M. Dorsch, Leah T. Vinson, Lisa A. Gunaydin

**Affiliations:** 1 Department of Psychiatry and Behavioral Sciences, University of California San Francisco, 675 Nelson Rising Lane, Room 314A, Box 0518, San Francisco, CA 94158 USA; 2 Department of Neurology, University of California San Francisco, San Francisco, CA 94158 USA; 3 Kavli Institute for Fundamental Neuroscience, University of California San Francisco, San Francisco, CA 94158 USA

**Keywords:** Emotion, Learning and memory, Neural circuits

## Abstract

Active avoidance behavior, in which an animal performs an action to avoid a stressor, is crucial for survival and may provide insight into avoidance behaviors seen in anxiety disorders. Active avoidance requires the dorsomedial prefrontal cortex (dmPFC), which is thought to regulate avoidance via downstream projections to the striatum and amygdala. However, the endogenous activity of dmPFC projections during active avoidance learning has never been recorded. Here we utilized fiber photometry to record from the dmPFC and its axonal projections to the dorsomedial striatum (DMS) and the basolateral amygdala (BLA) during active avoidance learning in both male and female mice. We examined neural activity during conditioned stimulus (CS) presentations and during clinically relevant behaviors such as active avoidance or cued freezing. Both prefrontal projections showed learning-related increases in activity during CS onset throughout active avoidance training. The dmPFC as a whole showed increased and decreased patterns of activity during avoidance and cued freezing, respectively. Finally, dmPFC-DMS and dmPFC-BLA projections show divergent encoding of active avoidance behavior, with the dmPFC-DMS projection showing increased activity and the dmPFC-BLA projection showing decreased activity during active avoidance. Our results demonstrate task-relevant encoding of active avoidance in projection-specific dmPFC subpopulations that play distinct but complementary roles in active avoidance learning.

## Introduction

Active avoidance, a behavioral coping strategy in which an organism performs an action to avoid a stressor, can be adaptively enacted to evade danger and ensure survival. However, active avoidance can become maladaptive when used in excess as seen in anxiety disorders. The dorsomedial prefrontal cortex (dmPFC) is an attractive candidate to explore in the context of active avoidance given its clear ties to anxiety disorder pathophysiology^1,2^ and avoidance behavior in humans^3–5^, as well as its roles in freezing, avoidance, and goal-directed behaviors in rodents^6–14^. Recent studies have revealed task-relevant neural activity in the dmPFC during platform-mediated avoidance and discriminative two-way active avoidance^10,14^, which inform our current understanding of the neural encoding of the dmPFC during active avoidance behavior. However, these studies have only examined neural activity on the final day of active avoidance training. Investigating how task-relevant signals in the dmPFC develop in real time across learning could help determine whether the dmPFC is preferentially recruited during certain stages of learning or whether task-relevant dmPFC activity is consolidated across days.

Further dissecting the dmPFC into subpopulations based on their projection target may also yield more refined insights into the nuanced and varied roles of the dmPFC in active avoidance behavior. One potential projection of interest is the dmPFC projection to the basolateral amygdala (BLA) given that the BLA has been consistently tied to active avoidance behavior^9,11,15–18^ and the dmPFC-BLA projection has an important role in other aversive learning paradigms such as fear conditioning and extinction^19,20^. The dmPFC-BLA projection has also been directly tied to active avoidance behavior, as optogenetically stimulating or inhibiting this projection bidirectionally affects platform-mediated active avoidance behavior^11^. While corticolimbic projections including the dmPFC-BLA projection have been more traditionally studied in the context of fear and avoidance behavior, recent evidence suggests that corticostriatal projections may also play a key role in avoidance behavior^21,22^. dmPFC projections to the dorsal striatum, especially the dorsomedial subregion (DMS), are uniquely positioned to play a crucial role in active avoidance behavior given their importance in goal-directed behavior^23–27^ and approach-avoidance decision making^21,22^. However, no studies have directly recorded the endogenous activity of either the dmPFC-BLA projection or dmPFC-DMS projection during active avoidance learning or expression.

In this study, we utilize fiber photometry in combination with retrograde viral targeting strategies to examine the activity of the dmPFC and its projections to the DMS and the BLA during learning and expression in a cued active avoidance task. As previous data from our laboratory has shown that the dmPFC-DMS and dmPFC-BLA projections are largely non-overlapping populations^21^, studying these discrete populations could provide novel insight into distinct dmPFC subpopulation functioning. We examined task-relevant neural activity in response to CS onset as well as clinically relevant behaviors such as avoidance and freezing. We find that dmPFC, the dmPFC-DMS projection, and the dmPFC-BLA projection show learning-related increases in activity at CS onset. However, encoding by the dmPFC-DMS and dmPFC-BLA projections diverges during avoidance onset, where we find increased activity in the dmPFC-DMS projection and decreased activity in the dmPFC-BLA projection. Finally, we identify decreases in dmPFC activity that correspond to freezing bouts. Overall, our results suggest that dmPFC and its projections to DMS and BLA contain task-relevant information and that through distinct encoding, the dmPFC-DMS and dmPFC-BLA projections may play complementary roles in successful enactment of active avoidance behavior.

## Materials and methods

All methods were carried out in accordance with relevant guidelines and regulations and are reported in accordance with ARRIVE guidelines for the reporting of animal experiments.

### Animals

We used male and female wild-type C57BL6/J mice 12–16 weeks of age purchased from Jackson Laboratories (4 dmPFC male, 6 dmPFC female, 4 dmPFC-DMS male, 4 dmPFC-DMS female, 5 dmPFC-BLA male, 4 dmPFC-BLA female). Animals were raised in normal light conditions (12:12 light/dark cycle) and given food and water ad libitum. All experimental protocols were approved by the Institutional Animal Care and Use Committee at the University of California, San Francisco. All experiments were conducted in accordance with procedures established by the Institutional Animal Care and Use Committee at the University of California, San Francisco. Sample size for dmPFC photometry was 10 mice, for dmPFC-DMS photometry was 8 mice, and for dmPFC-BLA photometry was 9 mice. Mice were excluded either due to no learning (2 dmPFC-DMS mice), improper targeting (1 dmPFC mouse, 1 dmPFC-DMS mouse), or low photometry signal (1 dmPFC mouse, 1 dmPFC-BLA mouse).

### Stereotaxic surgery, viral injections, and fiber optic cannula implantation

Surgeries were performed at 10–14 weeks of age. For fiber photometry, we injected 500 nL of AAV5-CaMKII-GCaMP6f into the dmPFC to record pyramidal neuron activity; to record dmPFC-DMS and dmPFC-BLA projection neurons, we injected 1500 nL of AAV1-Syn-Flex-GCaMP6m into the dmPFC and 500 nL of CAV2-Cre and hSyn-mCherry into the DMS and BLA. Injection coordinates (in millimeters relative to bregma) were as follows: dmPFC (1.8 A/P, −0.35 M/L, −2.4 D/V), DMS (0.8 A/P, −1.5 M/L, −3.5 D/V), BLA (−1.4 A/P, −3.3 M/L, −4.9 D/V). For all fiber photometry experiments, we implanted a 2.5 mm metal fiber optic cannula with 400 µm fiber optic stub (Doric Lenses, Quebec, Canada) in the dmPFC and waited 4–5 weeks for viral expression. Implant coordinates for the mPFC were 1.8 A/P, −0.35 M/L, −2.2 D/V. All viruses were obtained from Addgene, UNC Vector Core, or Institut de Génétique Moléculaire de Montpellier, Montpellier, France. See Supplemental Methods for detailed surgery and injection methods.

### Active avoidance behavior

Mice underwent a two-way active avoidance procedure adapted from a previous paper^18^. For our study, passive avoidance components of the original task were removed as we sought to focus solely on active avoidance. Active avoidance training occurred in a custom made apparatus consisting of two shock floors with strips of visible spectrum LED lights underneath each shock floor. Both shock and light presentations were controlled by an arduino using custom-made arduino code (Arduino, Somerville, MA, USA) in conjunction with location data from video recording software, Ethovision XT (Noldus, Wageningen, Netherlands). All trials were conducted in the dark and infrared lights beneath each shock floor were used to track the animals. Each day started with a 1 min baseline period followed by 30 active avoidance trials. Each active avoidance trial consisted of a 10 s light cue followed by 10 s of light plus 0.3 mA shock. Light and shock were presented on the shock floor the mouse was currently on at the initiation of the trial. Mice were able to avoid the shock altogether by moving onto the other unlit chamber during the 10 s light-only period. This was considered a successful active avoidance trial. Trials in which the mouse failed to move to the other unlit chamber during the 10 s of light-only are considered unsuccessful trials. There was a randomized intertrial interval of 20–30 s between each active avoidance trial. The light cue and shock cue lasted the allotted amount of time (20 s light, 10 s shock) regardless of the avoidance response. At the end of all 30 active avoidance trials there was a 1 min recovery period. All mice underwent 30 active avoidance trials per day for 5 days.

### Fiber photometry recording and data analysis

In vivo calcium data were acquired using a custom-built rig based on a previously described setup^28^. Raw photoreceiver data was extracted and signals were demodulated and normalized. Data was analyzed in PyCharm CE (JetBrains, Prague, Czechia) environment. Behavioral, location, and movement initiation data was extracted from both Ethovision and Arduino and synced to Synapse fiber photometry data. From this we extracted the behavioral data (percent avoidance, avoidance latency, and freezing) across all five days of learning. Additionally, we generated peri-event time histograms and heatmaps by time-locking the neural activity (dF/F) and z-scoring the signal to the baseline period (last 10 s of inter-trial-interval (ITI) preceding the event). These events included CS (light) onset (also split into successful and unsuccessful trials), avoidance movement initiation (movements during the 10 s light only period of successful trials), and freezing behavior initiation (freezing during the 10 s light only period of all trials). In addition, we also analyzed movement initiations during the ITI periods across all days. The heatmaps for avoidance movements and freezing were sorted by avoidance latency and freezing duration respectively. All other non-avoidance movement controls were quantified identically to avoidance movement. Lastly, histograms of the distribution of velocity and movement duration for all movement parameters were generated in Prism using a bin width of 1 cm/sec and 1 s respectively. See Supplemental Methods for quantification time windows.

### Movement and freezing behavior analysis

Following the recording of location data using Ethovision, post data collection analysis was performed to identify movement initiations using Ethovision’s built in movement detection software. The detection settings used were a 10 sample averaging window, 2.25 cm/sec start velocity threshold, and 2 cm/sec stop velocity threshold. Additionally, we used open source code^29^ to identify freezing. The parameters we used for this analysis were a motion cutoff of 9.0, freezing threshold of 1000, and minimum freeze duration of 25 samples (1 s).

### Statistical analysis

Statistical Analysis was performed with Prism 8 (Graphpad Software, San Diego, CA, USA). Normality was tested with D'Agostino & Pearson normality test. Paired t-test (two-tailed, assume gaussian distribution), one-way repeated measures ANOVA with Geisser-Greenhouse correction with Sidak’s and Tukey’s correction for multiple comparisons, and two-way repeated measures ANOVA with Sidak’s and Tukey’s correction for multiple comparisons (assume sphericity) was used.

### Data and code accessibility

The datasets used and/or analyzed during the current study are available from the corresponding author on reasonable request.

## Results

### dmPFC shows learning-related increases in activity at CS onset

**Figure 1:**
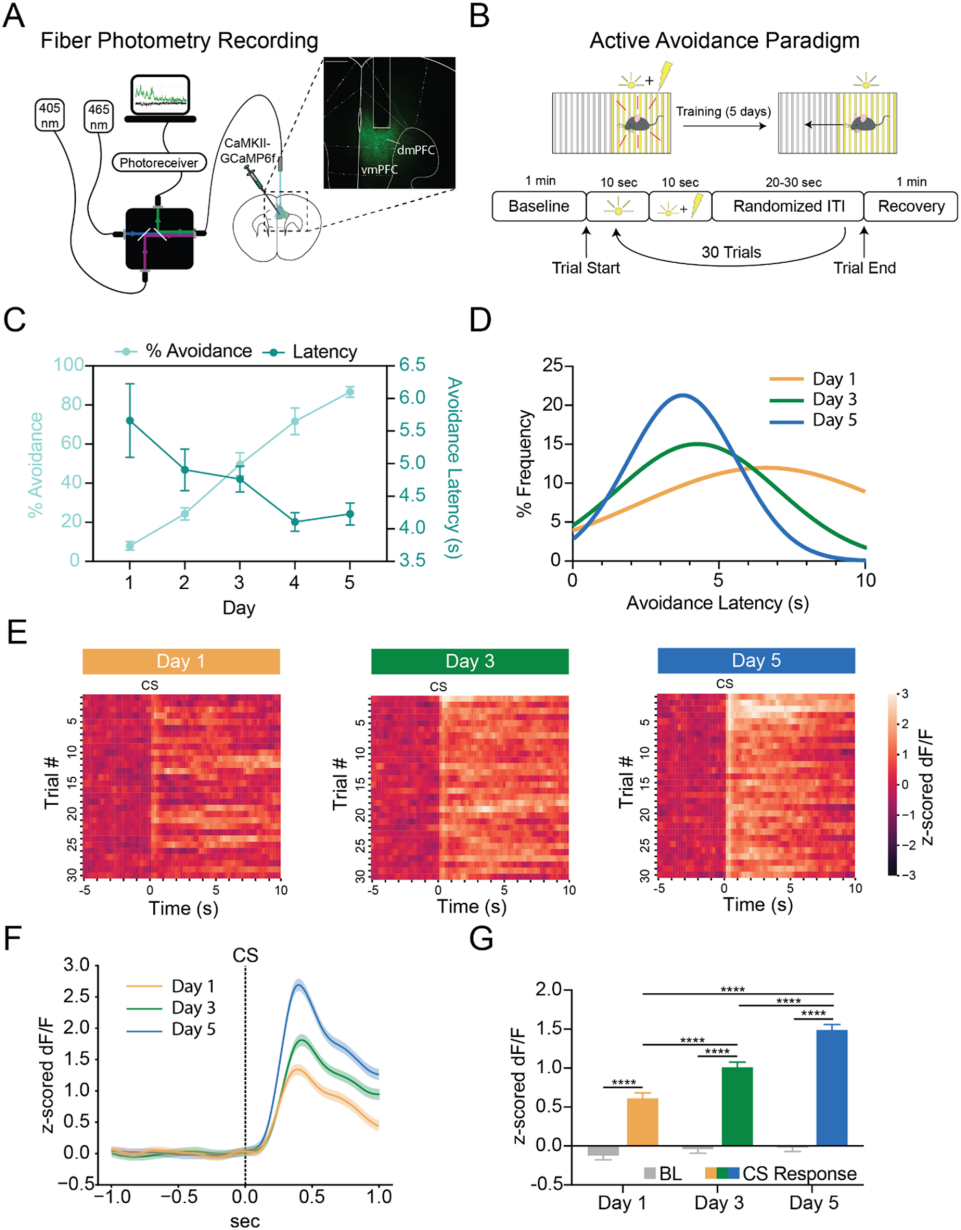
dmPFC shows learning-related increases in activity at CS onset during active avoidance learning. (**A**) Fiber photometry recording of dmPFC pyramidal neurons expressing GCAMP along with a representative histological image of GCAMP viral expression in the dmPFC. Scale bar 500 µm. (**B**) Behavioral schematic for active avoidance paradigm. (**C**) Average percent successful avoidance increased while avoidance latency decreased across training days. (**D**) Avoidance latency distribution shows avoidance latencies become shorter and more stereotyped across training. (**E**) Heatmaps of average change in calcium signal (z-scored dF/F) for each of the 30 trials presented in order from the first to the last trial for Day 1 (left), Day 3 (middle), and Day 5 (right). Heatmaps are aligned to CS onset (time zero) and show the total 10 s CS only period. dmPFC shows increased calcium signal at CS onset that becomes more consistent and sustained with training. (**F**) Perievent time histogram (PETH) showing increases in dmPFC calcium signal following CS onset. Orange line, mean ± standard error of the mean (SEM) for Day 1; green line, mean ± SEM for Day 3; blue line, mean ± SEM for Day 5. (**G**) Quantification of CS onset PETH shows calcium signal is significantly higher during the CS period (0 to 1 s) compared to the baseline period (−1 to 0 s) for all days. ****p < 0.0001.

We recorded the endogenous activity of excitatory dmPFC neurons during avoidance learning using a virally-expressed calcium indicator (GCaMP) and fiber photometry (Fig. 1A, Supplementary Figs. 1, 2). All mice were trained for 30 trials a day for five days on a cued two-way active avoidance behavioral paradigm (Fig. 1B). Average percent successful avoidance increased across training and did not differ based on sex (Fig. 1C, Supplementary Fig. 3). Average avoidance latency (between 4 and 6 s) decreased across training and became more stereotyped (Fig. 1C,D). Heatmaps of the average change in calcium signal in the dmPFC for each trial during the CS-only period (first 10 s of the CS before the shock occurred) for days 1, 3, and 5 showed a rapid peak in fluorescence at CS onset as well as a sustained increase in fluorescence that appeared to develop across learning (Fig. 1E). A perievent time histogram (PETH) of the z-scored change in dmPFC calcium signal for the first second of the CS presentation (CS onset specific neural activity, > 90% avoidances occur after this time window) found that the dmPFC showed a sharp increase in fluorescence during the first second of CS onset compared to the baseline period; this effect was significant on all training days. However, the magnitude of the increase in fluorescence significantly increased across days (Fig. 1F,G, Two-way ANOVA, Training Day × Task Period F_(2, 1794)_ = 21.74, *p* < 0.0001, Training Day *p* < 0.0001, Task Period *p* < 0.0001; Sidak’s Multiple Comparisons Test, Day 1 Baseline vs Day 3 Baseline *p* = 0.9949, Day 1 Baseline vs Day 5 Baseline *p* = 0.9684, Day 1 Baseline vs Day 1 CS *p* < 0.0001, Day 1 CS vs Day 3 CS p < 0.0001, Day 1 CS vs Day 5 CS *p* < 0.0001, Day 3 Baseline vs Day 5 Baseline *p* > 0.9999, Day 3 Baseline vs Day 3 CS *p* < 0.0001, Day 3 CS vs Day 5 CS *p* < 0.0001, Day 5 Baseline vs Day 5 CS *p* < 0.0001; N = 10 mice, n = 300 trials). dmPFC neural activity at CS onset positively correlated with percent avoidance across training (Supplemental Figure 4). There were no significant differences between successful and unsuccessful trials during the first second after CS onset and no significant within-day differences in the amplitude of the dmPFC calcium signal (Supplemental Figures 4, 5). Taken together, these data suggest that there are learning-related increases in neural activity in the dmPFC during CS onset that become amplified across active avoidance learning.

### dmPFC shows opposing patterns of activity during active avoidance and cued freezing

**Figure 2:**
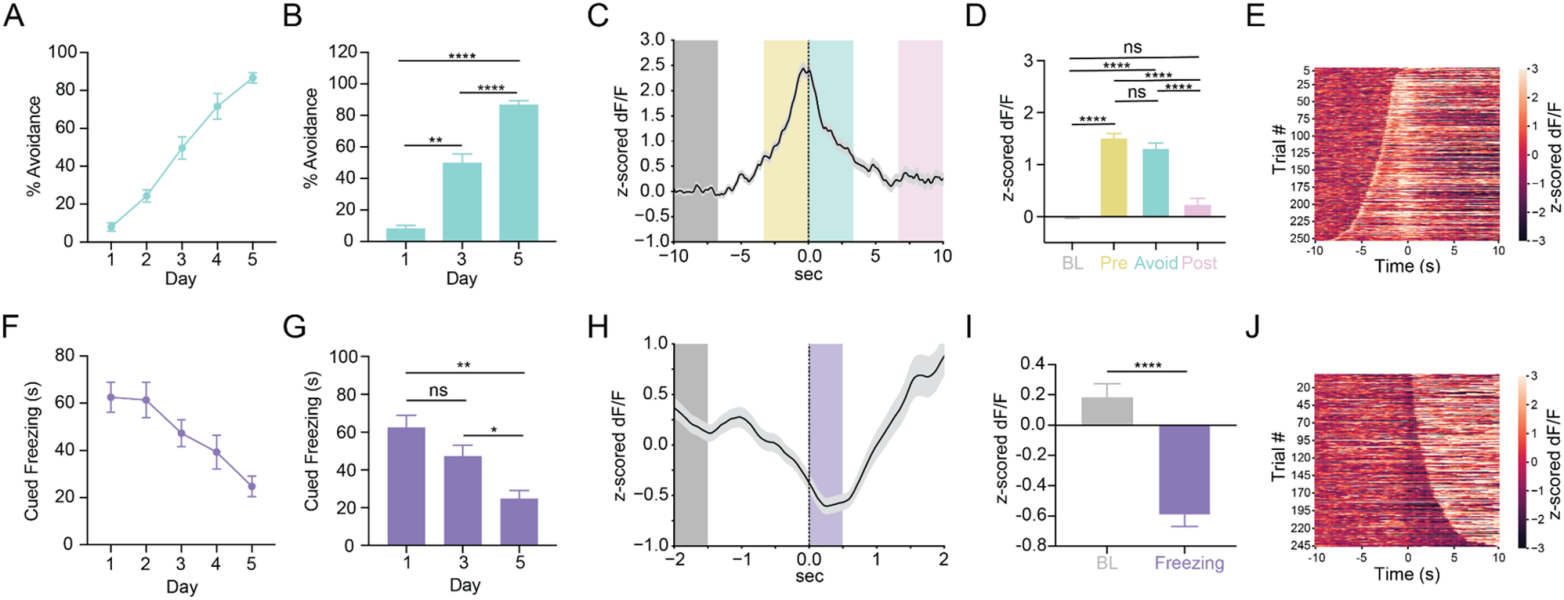
dmPFC shows opposing patterns of activity during active avoidance and cued freezing behavior. (**A**) Percent successful avoidance across training days. Learning curve displays the same data as seen in Fig. 1C. (**B**) Quantification of percent successful avoidance shows animals significantly increase avoidance across training. (**C**) PETH shows an increase in calcium signal at avoidance onset on Day 5. Line with shading represents mean ± SEM. Grey box, baseline period (BL); yellow box, pre-avoidance period (Pre); teal box, avoidance period (Avoid); pink box, post-avoidance period (Post). (**D**) Quantification of avoidance PETH reveals significantly increased calcium signal in the pre-avoid (−3 to 0 s) and avoid (0 to 3 s) periods but not the post-avoid period (7 to 10 s) compared to the baseline period (−10 to −7 s). (**E**) Heatmap of change in calcium signal for all avoidance trials for all mice from Day 5 aligned to avoidance onset and sorted from shortest to longest avoidance latency. Heatmap shows distinct increases in calcium signal at CS onset (slope curving leftward) and avoidance onset (time zero). (**F**) Cued freezing during the CS-only period across training days. (**G**) Quantification of cued freezing shows that animals significantly decrease cued freezing across training. (**H**) PETH shows decrease in calcium signal at freezing onset on Day 1. Line with shading represents mean ± SEM. Grey box, baseline period (BL); Purple box, freezing period (Freezing). (**I**) Quantification of freezing PETH shows significant decrease in calcium signal during the freezing period (0–0.5 s) compared to the baseline period (−2 to −1.5 s). (**J**) Heatmap of change in calcium signal during all individual freezing bouts from all mice from Day 1 aligned to freezing onset and sorted from shortest to longest freezing bout. Heatmap shows dips in calcium signal at freezing onset that increases in length as freezing bout duration increases. ns = not significant, *p < 0.05, **p < 0.01, ****p < 0.0001.

We next sought to examine dmPFC neural activity during active avoidance and freezing behaviors on the day where each behavior was most prevalent (Day 5 for avoidance and Day 1 for freezing). Due to limited data, we do not make conclusions about changes in signal across days. The number of successful avoidances significantly increased across learning (Fig. 2A,B, Repeated Measures One-way ANOVA F_(1.2, 10.8)_ = 165.9, *p* < 0.0001; Sidak’s Multiple Comparisons Test, Day 1 vs Day 3 *p* = 0.0002, Day 1 vs Day 5 *p* < 0.0001, Day 3 vs Day 5 *p* < 0.0001; N = 10 mice). When aligning the dmPFC calcium signal to avoidance onset on day 5 (Fig. 2C), we found a statistically significant increase in fluorescence during the avoidance period compared to the baseline period (Fig. 2D, Supplemental Figure 6, Repeated Measures One-Way ANOVA F_(2.549, 642.4)_ = 114.1, *p* < 0.0001; Tukey’s Multiple Comparisons Test, Baseline vs Pre Avoid *p* < 0.0001, Baseline vs Avoid *p* < 0.0001, Baseline vs Post Avoid *p* = 0.1731, Pre Avoid vs Avoid *p* = 0.0586, Pre Avoid vs Post Avoid *p* < 0.0001, Avoid vs Post Avoid *p* < 0.0001; N = 10 mice, n = 253 trials). We found significantly increased fluorescence in the avoidance movement trace during the pre-movement and/or movement periods compared to ITI movement traces of similar velocity or duration from the same recording day, suggesting that the increase in calcium signal during avoidance movements was not purely movement-related (Supplemental Figure 7). Heatmaps of calcium activity for all individual avoidance trials on Day 5 aligned to avoidance onset and sorted from shortest to longest avoidance latency showed a consistent time-locked peak in fluorescence corresponding to avoidance onset and a sharp moving peak of fluorescence likely representing the increase in calcium signal at CS onset (Fig. 2E). These data suggest that the dmPFC separately encodes both the CS onset and avoidance onset through distinct increases in neural activity.

In contrast to avoidance, freezing during the CS-only period (cued freezing) significantly decreased across learning (Fig. 2F,G, Repeated Measures One-way ANOVA F_(1.441,, 12.97)_ = 9.829, *p* = 0.0045; Sidak’s Multiple Comparisons Test, Day 1 vs Day 3 *p* = 0.4807, Day 1 vs Day 5 *p* = 0.0024, Day 3 vs Day 5 *p* = 0.023; N = 10 mice). Cued freezing was evenly distributed before and after avoidance behavior as well as throughout the CS-only period. There were no significant differences in cued freezing between successful and unsuccessful trials (Supplemental Figure 8). A PETH of dmPFC calcium activity aligned to freezing onset on day 1 for all cued freezing bouts with a 1 s minimum duration (Fig. 2H) showed a statistically significant decrease in fluorescence during the freezing period compared to the baseline period (Fig. 2I, Supplemental Figure 6, Paired t-test t = 9.603, df = 245, *p* < 0.0001; N = 10 mice, n = 246 trials). A heatmap of calcium activity on all individual trials for day 1 aligned to freezing onset and sorted by shortest to longest freezing bout duration showed a dip in fluorescence at freezing onset that increased in duration with longer freezing bouts (Fig. 2J). Overall, our results suggest that the dmPFC shows opposing patterns of activity during avoidance and freezing and that these patterns of activity are distinct from the neural activity observed during CS onset.

### dmPFC-DMS and dmPFC-BLA show learning-related increases in activity at CS onset

**Figure 3:**
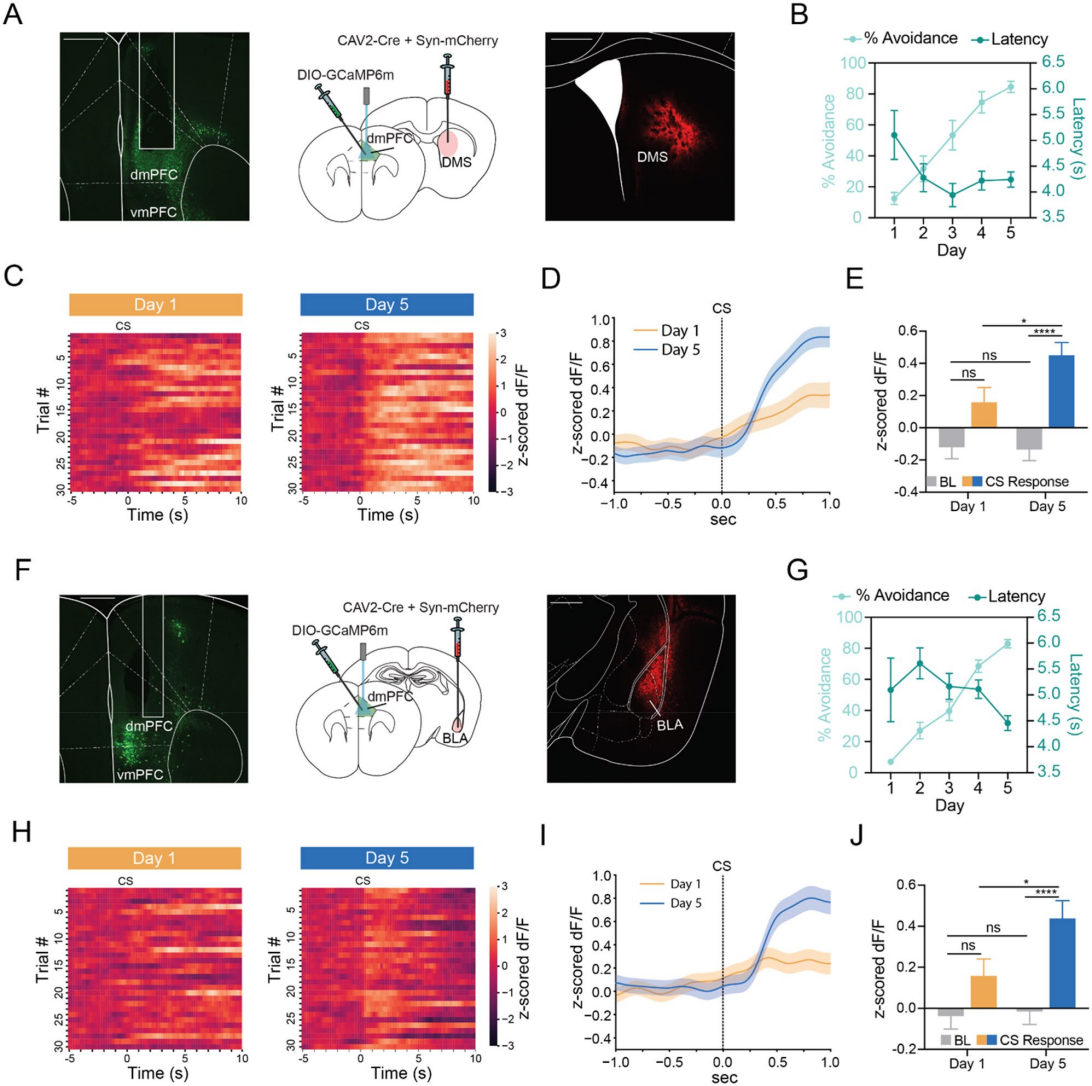
dmPFC-DMS and dmPFC-BLA projections show similar learning-related increases in activity at CS onset during active avoidance learning. (**A**) Viral targeting strategy for dmPFC-DMS photometry along with representative histological image of fiber photometry implant and GCAMP viral expression in dmPFC (left) and CAV2-Cre + mCherry viral expression in the DMS (right). Scale bar 500 µm. (**B**) Percent avoidance increases while avoidance latency decreases across training in the dmPFC-DMS cohort. (**C**) Heatmaps of change in calcium signal aligned to CS onset for each of the 30 trials arranged from first to the last trial for Day 1 (left) and Day 5 (right). dmPFC-DMS projection shows sustained increases in calcium signal at CS onset that become more consistent across training. (**D**) PETH shows increases in signal at CS onset in the dmPFC-DMS projection following training. orange line, mean ± SEM for Day 1; blue line, mean ± SEM for Day 5. (**E**) Quantification of the dmPFC-DMS CS onset PETH shows significant increase in calcium signal during the CS period (0 to 1 s) compared to the baseline period (-1 to 0 s) for Day 5, but not Day 1. (**F**) Viral targeting strategy for dmPFC-BLA photometry along with representative histological image of fiber photometry implant and GCAMP viral expression in dmPFC (left) and CAV2-Cre + mCherry viral expression in the BLA (right). Scale bar 500 µm. (**G**) Percent avoidance increases while avoidance latency decreases across training in the dmPFC-BLA cohort. (**H**) Heatmaps of change in calcium signal aligned to CS onset for each of the 30 trials arranged from first to the last trial for Day 1 (left) and Day 5 (right). dmPFC-BLA projection shows transient increases in calcium signal at CS onset only during later stages of training. (**I**) PETH shows increases in signal at CS onset in the dmPFC-BLA projection following training. orange line, mean ± SEM for Day 1; blue line, mean ± SEM for Day 5. (**J**) Quantification of the dmPFC-BLA CS onset PETH shows significant increase in calcium signal during the CS period (0 to 1 s) compared to the baseline period (−1 to 0 s) for Day 5, but not Day 1. ns = not significant, *p < 0.05, ****p < 0.0001.

We next obtained projection-specific fiber photometry recordings from the dmPFC-DMS projection using a dual virus retrograde targeting strategy (Fig. 3A, Supplemental Figure 9). Mice learned to successfully avoid 80% of the time by day 5 and average avoidance latencies (between 4 and 6 s) decreased across training (Fig. 3B, Supplemental Figure 10). Heatmaps of dmPFC-DMS average change in calcium signal for each trial during the CS-only period for day 1 and day 5 revealed a sustained increase in fluorescence during the CS-only period that became time locked to CS onset and more consistent as training progressed (Fig. 3C). Additionally, a PETH of the z-scored change in dmPFC-DMS calcium signal for the first second of the CS presentation showed a significant increase in calcium signal at CS onset compared to baseline on day 5 but not on day 1 in addition to a significant increase in calcium signal at CS onset across days (Fig. 3D,E, Two-way ANOVA, Training Day × Task Period F_(1, 1196)_ = 3.856, *p* = 0.0498, Training Day p = 0.0725, Task Period *p* < 0.0001; Sidak’s Multiple Comparisons Test, Day 1 Baseline vs Day 1 CS *p* = 0.0634, Day 1 Baseline vs Day 5 Baseline *p* > 0.9999, Day 1 CS vs Day 5 CS *p* = 0.0466, Day 5 Baseline vs Day 5 CS *p* < 0.0001; N = 8 mice, n = 300 trials).

We next examined neural activity in the dmPFC-BLA projection during active avoidance learning using the same retrograde viral targeting strategy (Fig. 3F, Supplemental Fig. 9). Behaviorally, we saw similar trends to the dmPFC-DMS cohort overall with there being no significant differences in the learning curve or in avoidance latencies on day 1 and day 5 between the dmPFC-DMS and the dmPFC-BLA cohorts (Fig. 3G, Supplemental Fig. 10). Heatmaps of the dmPFC-BLA average change in calcium signal for each trial during the CS-only period for day 1 and day 5 showed no organized pattern of fluorescence on day 1, but a clear transient increase in fluorescence time locked to CS onset on day 5 (Fig. 3H). A PETH of the z-scored change in dmPFC-BLA calcium signal for the first second of the CS presentation showed a significant increase in calcium signal at CS onset compared to baseline on day 5 but not on day 1 in addition to a significant increase in calcium signal at CS onset across days (Fig. 3I,J, Two-way ANOVA, Training Day × Task Period F_(1,1196)_ = 3.038, *p* = 0.0816, Training Day *p* = 0.0411, Task Period *p* < 0.0001; Sidak’s Multiple Comparisons Test, Day 1 Baseline vs Day 5 Baseline *p* > 0.9999, Day 1 Baseline vs Day 1 CS *p* = 0.3023, Day 1 CS vs Day 5 CS *p* = 0.0442, Day 5 Baseline vs Day 5 CS *p* < 0.0001; N = 9 mice, n = 300 trials). Both dmPFC-DMS and dmPFC-BLA neural activity at CS onset positively correlated with percent avoidance across training and CS-evoked fluorescence changes during successful trials did not significantly differ from that on unsuccessful trials for either projection (Supplemental Fig. 11). Overall, our results suggest that both the dmPFC-DMS and dmPFC-BLA projections show learning-related increases in neural activity at CS onset during active avoidance learning.

### dmPFC-DMS and dmPFC-BLA show divergent encoding of active avoidance behavior

**Figure 4:**
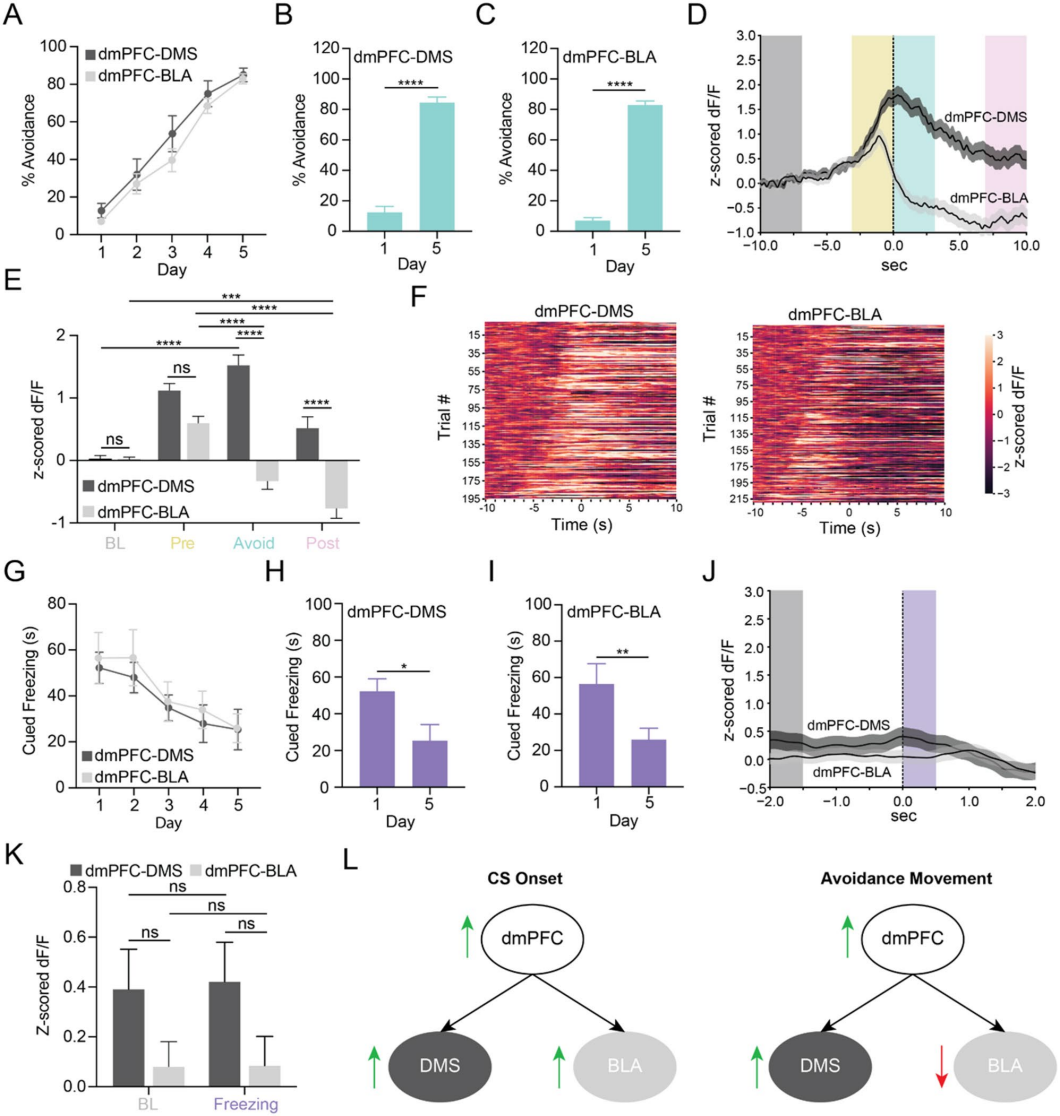
dmPFC-DMS and dmPFC-BLA projections show divergent encoding of active avoidance behavior. (**A**) Percent avoidance across training days in the dmPFC-DMS (dark grey line) and dmPFC-BLA (light grey line) cohort. (**B**, **C**) Percent avoidance significantly increases from Day 1 to Day 5 in the dmPFC-DMS (left) and dmPFC-BLA (right) cohort. (**D**) PETH shows increase in calcium signal in the dmPFC-DMS projection and decrease in calcium signal in the dmPFC-BLA projection during avoidance onset on Day 5. Dark grey line, mean ± SEM for dmPFC-DMS projection; light grey line, mean ± SEM for dmPFC-BLA projection; Grey box, baseline period (BL); yellow box, pre-avoidance period (Pre); teal box, avoidance period (Avoid); pink box, post-avoidance period (Post). (**E**) Quantification of avoidance PETH shows a significant increase in calcium signal in the avoid (0 to 3 s) period compared to baseline period (-10 to -7 s) for dmPFC-DMS projection. The dmPFC-BLA projection shows a significance decrease in signal during the avoid (0 to 3 s) period compared to the pre-avoid period (−3 to 0 s) and a significance decrease in signal during the post-avoid (7 to 10 s) period compared to the baseline (−10 to −7 s) and pre-avoid (−3 to 0 s) periods. (**F**) Heatmap of change in calcium signal for all individual avoidance trials for all mice from Day 5 aligned to avoidance onset and sorted from shortest to longest avoidance latency for the dmPFC-DMS (left) and dmPFC-BLA (right) projections. (**G**) Cued freezing in the dmPFC-DMS (dark grey line) and the dmPFC-BLA (light grey line) cohort. (**H**, **I**) Cued freezing significantly decreases from Day 1 to Day 5 in the dmPFC-DMS (left) and the dmPFC-BLA (right) cohorts. (**J**) PETH shows no change in calcium signal at freezing onset for either the dmPFC-DMS or the dmPFC-BLA projection on Day 1. Dark grey line, mean ± SEM for dmPFC-DMS projection; light grey line, mean ± SEM for dmPFC-BLA projection; Grey box, baseline period (BL); Purple box, freezing period (Freezing). (**K**) Quantification of freezing PETH shows no significant change in calcium signal during the freezing period (0–0.5 s) compared to the baseline period (−2 to −1.5 s). (**L**) Graphical abstract summarizing main findings from the study. ns = not significant, *p < 0.05, **p < 0.01, ***p < 0.001, ****p < 0.0001.

We were additionally interested in examining projection-specific neural activity during avoidance and freezing behaviors. Both cohorts reached 80% successful avoidance by day 5 of learning (Fig. 4A–C, dmPFC-DMS Paired t-test t = 25.01, df = 7, *p* < 0.0001, dmPFC-BLA Paired t-test t = 4.161, df = 8, *p* < 0.0001; dmPFC-DMS N = 8 mice, dmPFC-BLA N = 9 mice). In a PETH aligned to avoidance onset, we found that the dmPFC-DMS projection showed a hill-like increase in fluorescence at avoidance onset while the dmPFC-BLA projection showed a descending slope (Fig. 4D). Validating these contrasting results, the dmPFC-DMS projection showed a significant increase in signal during the avoidance period compared to the baseline period while the dmPFC-BLA projection showed a significant decrease in signal during the avoidance period compared to the pre-avoidance period and during the post-avoidance period compared to the baseline and pre-avoidance period. In addition, the dmPFC-DMS and the dmPFC-BLA calcium signals statistically differed from each other during the avoidance and post-avoidance periods (Fig. 4E, Supplemental Fig. 12, Two-way ANOVA, Task Period × Projection F_(3, 1616)_ = 21.65, *p* < 0.0001, Task Period *p* < 0.0001, Projection *p* < 0.0001; Sidak’s Multiple Comparisons Test, dmPFC-DMS Baseline vs dmPFC-DMS Avoid *p* < 0.0001, dmPFC-BLA Baseline vs dmPFC-BLA Post Avoid *p* = 0.0002, dmPFC-BLA Pre Avoid vs dmPFC-BLA Avoid *p* < 0.0001, dmPFC-BLA Pre Avoid vs dmPFC-BLA Post Avoid *p* < 0.0001, dmPFC-DMS Baseline vs dmPFC-BLA Baseline *p* > 0.9999, dmPFC-DMS Pre Avoid vs dmPFC-BLA Pre Avoid *p* = 0.0836, dmPFC-DMS Avoid vs dmPFC-BLA Avoid *p* < 0.0001, dmPFC-DMS Post Avoid vs dmPFC-BLA Post Avoid *p* < 0.0001; dmPFC-DMS N = 8 mice, n = 195 trials, dmPFC-BLA N = 9 mice, n = 211 trials). We found significant differences in fluorescence between avoidance movements and ITI movements of similar duration, suggesting that the changes in calcium activity in these projections during avoidance onset were not purely movement-related (Supplemental Fig. 13). A heatmap of dmPFC-DMS calcium activity on all individual trials on day 5 aligned to avoidance onset and sorted from shortest to longest avoidance latency showed an increase in fluorescence curving leftwards that likely corresponds to CS onset with no clear distinctions in signal between when the CS began and when the avoidance began. In contrast, in the dmPFC-BLA projection heatmap, there was a clear increase in fluorescence sloping leftward that likely corresponded to CS onset, whereas avoidance onset was marked by a time-locked drop in fluorescence (Fig. 4F).

We next examined how the dmPFC-DMS and the dmPFC-BLA projections encoded freezing behavior, and found statistically significant decreases in freezing on day 5 compared to day 1 for each projection (Fig. 4G–I, dmPFC-DMS Paired t-test t = 2.387, df = 7, *p* = 0.0484, dmPFC-BLA Paired t-test t = 4.161, df = 8, *p* = 0.0032; dmPFC-DMS N = 8 mice, dmPFC-BLA N = 9 mice). However, there was no significant difference in signal between the baseline period and the freezing period in the perievent time histograms aligned to freezing onset for each projection (Fig. 4J,K, Supplemental Fig. 12, Two-way ANOVA, Task Period × Projection F_(1, 820)_ = 0.009249, *p* = 0.9234, Task Period *p* = 0.8965, Projection *p* = 0.0145; Sidak’s Multiple Comparisons Test, dmPFC-DMS Baseline vs dmPFC-BLA Baseline *p* = 0.4562, dmPFC-DMS Baseline vs dmPFC-DMS Freezing *p* > 0.9999, dmPFC-BLA Baseline vs dmPFC-BLA Freezing *p* > 0.9999, dmPFC-DMS Freezing vs dmPFC-BLA Freezing 0.3624; dmPFC-DMS N = 8 mice, n = 183 trials, dmPFC-BLA N = 9 mice, n = 229 trials). Overall, our results show opposing patterns of activity in the dmPFC-DMS and dmPFC-BLA projection during active avoidance behavior, with increased activity in the dmPFC-DMS projection and decreased activity in the dmPFC-BLA projection at avoidance onset. The main findings from our study are summarized in Fig. 4L.

## Discussion

We found that the dmPFC and its projections to the DMS and the BLA contain learning-related increases in activity at CS onset during active avoidance. Encoding of active avoidance diverged in the dmPFC-DMS and dmPFC-BLA projections, which showed increased and decreased neural activity at avoidance onset, respectively. To our knowledge, this is the first study to record the endogenous activity of distinct dmPFC projections during active avoidance behavior. Our results reveal the importance of studying projection-defined dmPFC subpopulations as they may reveal distinct circuit encoding which supports active avoidance learning and expression.

The task-relevant neural encoding in the dmPFC during two-way active avoidance behavior found in this study is of note as recent literature has shown differing contributions of the dmPFC to active avoidance behavior depending on the task design used. dmPFC has been implicated in active avoidance tasks which involve decision making under conflict (platform-mediated avoidance)^9–11^, tasks which require discrimination between a CS + predictive of shock and CS- not predictive of shock (discriminative two-way active avoidance)^14^, or in which both active and passive avoidance components are present concurrently^13^. However, previous studies have found mixed results regarding the importance of the dmPFC in active avoidance behavioral paradigms in which one action such as lever press (lever press avoidance)^12,13^ or shuttling (two-way active avoidance)^30^ is triggered by a single cue. However, these studies utilized lesions, which could lead to network compensation prior to task learning, or muscimol, which inactivates the entire region and cannot decipher the contributions of distinct subpopulations of cells within a given region. Thus, although previous studies have not consistently implicated the dmPFC as a whole in two-way active avoidance, we found that projection-defined excitatory dmPFC subpopulations do show robust task-relevant encoding of active avoidance behavior during a two-way active avoidance task, a finding which may have been obscured in previous studies due to differences in the specificity of techniques utilized. We compare the findings in our study here to previous studies which have examined neural encoding in the dmPFC during platform-mediated avoidance and discriminative two-way active avoidance, although we note that differences in task designs limit parallels which can be drawn.

The dmPFC as a whole showed learning-related increases in activity at CS onset. Given that significant differences in neural activity at CS onset were seen across days but not within days suggests that the learning-related increase in activity at CS onset in the dmPFC is a consolidated phenomenon that gradually builds across time. Recent studies have also found task-relevant CS activity in the dmPFC during platform-mediated avoidance and discriminative two-way active avoidance^10,14^. However, results from the platform-mediated avoidance task showed inhibition of single dmPFC units upon CS onset unique to avoidance rather than the increases in activity as seen in our two-way active avoidance task^10^. This difference in signal could be explained by key differences in the tasks used. The platform-mediated avoidance task seeks to model anxiety through using approach-avoidance decision making or decision making under conflict as rodents must choose whether to obtain reward or forgo reward to avoid shock. Our two-way active avoidance task instead seeks solely to model avoidance behavior, only involving shock and as such has no conflict component. Studies have shown that the dmPFC differently encodes situations involving decision making under conflict versus no conflict^22^, which could explain the difference in encoding in the dmPFC seen between these two tasks. Additionally, there are technical differences between bulk calcium recording (photometry) and single unit electrophysiology that could explain differences in the results seen. Photometry records a summed signal of activity instead of reflecting single units. In the platform-mediated avoidance task, there were a greater number of cells excited by the CS than inhibited and thus, we could be picking up the bulk sum which would result in net excitation. Additionally, calcium indicators are more sensitive to increases rather than decreases in activity^31^, and thus may preferentially detect excitation rather than inhibition. However, photometry was our technique of choice given that a major goal of our study was to identify neural activity in projection-defined subpopulations of dmPFC neurons, which are not as easily differentiated by single unit in vivo electrophysiological recordings. Finally, we targeted more caudal regions of dmPFC whereas the inhibitory signal was seen in rostral dmPFC in the platform-mediated avoidance task, thus differences in the regions targeted may also contribute to differences in the signal seen. While there were differences in the directionality of the signal seen in the platform-mediated avoidance task and our two-way active avoidance task, our study also found no difference in CS-evoked neural signal between successful and unsuccessful trials, which is an observation supported by both platform-mediated and additional discriminative two-way active avoidance studies^10,14^. This result could suggest that the CS-evoked activity may signal the option to avoid rather than the avoidance behavior itself^10^. Overall, this is the first study to our knowledge to examine longitudinal learning-related changes in dmPFC activity across days of an active avoidance task.

The dmPFC also showed opposing patterns of activity during avoidance and freezing behavior, with increases in activity during avoidance and decreases in activity during freezing. Previous studies have also shown increased activity in the dmPFC during both platform-mediated avoidance and discriminative two-way active avoidance^10,14^, however there has been conflict as to whether to attribute this increase in activity to avoidance behavior specifically or to movement more generally. In our study, we compare dmPFC neural activity during avoidance movements and intertrial interval movements of similar duration and velocity and find that the increased neural activity seen during avoidance is not accounted for by general movement alone. This finding is corroborated by another study using dmPFC activity to decode avoidance behavior in a discriminative two-way active avoidance task, which found an increase in decoding accuracy within the last second before the avoidance movement which could not be accounted for by speed^14^. Both of these findings suggest that the increased activity seen at avoidance onset during two-way active avoidance tasks may encode aspects unique to avoidance rather than solely encoding movement more generally. The decrease in dmPFC activity we see during freezing is in contrast with in vivo electrophysiology studies which have found increased firing rates in dmPFC neurons during freezing behavior in classical and discriminative fear conditioning tasks^32–34^. Given that calcium indicators are more sensitive to increases rather than decreases in activity^31^, this difference seems likely unrelated to technique used and may instead be due to key differences in the tasks, such as the fact that the active avoidance task allows for both passive and active coping responses to threat, whereas in classical fear conditioning animals have no control over the shocks and therefore are biased toward passive coping via freezing.

Both the dmPFC-DMS and dmPFC-BLA projections showed learning-related increases in activity at CS onset and there were no differences in activity between successful and unsuccessful trials. Interestingly, although there was a decrease in dmPFC activity during freezing behavior, neither the dmPFC-DMS projection nor the dmPFC-BLA projection encoded freezing behavior. Potential candidate projections which may contain dmPFC-specific encoding of freezing behavior could include the dmPFC projection to the paraventricular thalamus (PVT) or the dmPFC projection to the periaqueductal grey (PAG), as optogenetic manipulations of both of these pathways has been shown to causally influence freezing behavior^35,36^.

While CS-aligned activity looked similar in both dmPFC projections, they displayed opposing patterns of activity at avoidance onset, with the dmPFC-DMS projection showing increased activity and the dmPFC-BLA projection showing decreased activity. The dmPFC-DMS projection directly interfaces downstream with the striatum which regulates motor control and action selection^37^ and is therefore poised to play a privileged role in aiding avoidance movement initiation. On the other hand, the dmPFC-BLA projection could influence BLA cells projecting to the central lateral amygdala (CeL), a region which has been shown to gate active and passive defensive responses^38^. Thus, the dmPFC-BLA projection could influence activity in downstream regions such as the CeL in favor of active defensive behaviors such as active avoidance over passive defensive behaviors such as freezing. As a result, the distinct neural activity in the dmPFC-DMS and dmPFC-BLA projections may play complementary roles in coordinating successful active avoidance behavior.

While the dmPFC-DMS projection has not been previously explored within the context of active avoidance, stimulation of the dmPFC-BLA projection increases avoidance in the platform-mediated avoidance task^11^. Our photometry results would suggest that inhibiting the dmPFC-BLA projection may increase avoidance in the two-way active avoidance task given that dmPFC-BLA activity decreases acutely during avoidance in our task. However, future optogenetic studies would be needed to confirm this hypothesis. In a previous study from our lab examining the dmPFC-DMS and dmPFC-BLA projections during an innate approach-avoidance task, we found that the dmPFC-DMS projection recapitulated whole population dmPFC activity while the dmPFC-BLA projection did not^21^. Similarly, here we find that the dmPFC-DMS projection shows increased activity during avoidance similar to the dmPFC overall, while the dmPFC-BLA projection shows distinct decreases in activity during avoidance. The projection-specific activity we observed during avoidance intriguingly parallels fMRI findings during active avoidance in humans which show increased coupling between mPFC and caudate (the human equivalent of the DMS) or mPFC and amygdala predicted better active avoidance performance while the caduate showed increased activity and the amygdala showed decreased activity during active avoidance behavior^4^. These results highlight the importance of mPFC communication with both the dorsal striatum and the amygdala and suggest conservation of function across species in these circuits during active avoidance behavior.

Overall, we find task-relevant information encoding in the dmPFC and its projections to the DMS and the BLA during active avoidance learning. We also find opposing patterns of activity in the dmPFC-DMS and dmPFC-BLA projections during active avoidance behavior, suggesting that these projections may play complementary roles in the successful enactment of active avoidance behavior through distinct neural encoding. Our findings, in combination with future research examining these projections during active avoidance in psychiatric disease models, may provide crucial first steps to identifying novel treatment targets to alleviate avoidance symptoms seen in anxiety disorders.

## Supplementary Information

Supplementary Information.

## References

[1] Holzschneider K, Mulert C (2011). Neuroimaging in anxiety disorders. Dialogues Clin. Neurosci..

[2] Rauch, S. L. & Shin, L. M. Structural and functional imaging of anxiety and stress disorders. in *Neuropsychopharmacology: The Fifth Generation of Progress* 953–966 (2002).

[3] Delgado MR, Jou RL, Ledoux JE, Phelps EA (2009). Avoiding negative outcomes: tracking the mechanisms of avoidance learning in humans during fear conditioning. Front. Behav. Neurosci..

[4] Collins KA, Mendelsohn A, Cain CK, Schiller D (2014). Taking action in the face of threat: neural synchronization predicts adaptive coping. J. Neurosci..

[5] Sripada RK, Garfinkel SN, Liberzon I (2013). Avoidant symptoms in PTSD predict fear circuit activation during multimodal fear extinction. Front. Hum. Neurosci..

[6] Giustino TF, Maren S (2015). The role of the medial prefrontal cortex in the conditioning and extinction of fear. Front. Behav. Neurosci..

[7] Tovote P, Fadok JP, Lüthi A (2015). Neuronal circuits for fear and anxiety. Nat. Rev. Neurosci..

[8] Gourley SL, Taylor JR (2016). Going and stopping: dichotomies in behavioral control by the prefrontal cortex. Nat. Neurosci..

[9] Bravo-Rivera C, Roman-Ortiz C, Brignoni-Perez E, Sotres-Bayon F, Quirk GJ (2014). Neural structures mediating expression and extinction of platform-mediated avoidance. J. Neurosci..

[10] Diehl, M. M. *et al.* Active avoidance requires inhibitory signaling in the rodent prelimbic prefrontal cortex. *Elife* **7**, (2018). 10.7554/eLife.34657 PMC5980229 29851381

[11] Diehl, M. M. *et al.* Divergent projections of the prelimbic cortex bidirectionally regulate active avoidance. *Elife* **9**, (2020). 10.7554/eLife.59281 PMC7588229 33054975

[12] Beck KD (2014). ITI-signals and prelimbic cortex facilitate avoidance acquisition and reduce avoidance latencies, respectively, in male WKY rats. Front. Behav. Neurosci..

[13] Capuzzo G, Floresco SB (2020). Prelimbic and infralimbic prefrontal regulation of active and inhibitory avoidance and reward-seeking. J. Neurosci..

[14] Jercog D (2021). Dynamical prefrontal population coding during defensive behaviours. Nature.

[15] Lázaro-Muñoz G, LeDoux JE, Cain CK (2010). Sidman instrumental avoidance initially depends on lateral and basal amygdala and is constrained by central amygdala-mediated Pavlovian processes. Biol. Psychiatry.

[16] Choi J-S, Cain CK, LeDoux JE (2010). The role of amygdala nuclei in the expression of auditory signaled two-way active avoidance in rats. Learn. Mem..

[17] Darvas M, Fadok JP, Palmiter RD (2011). Requirement of dopamine signaling in the amygdala and striatum for learning and maintenance of a conditioned avoidance response. Learn. Mem..

[18] Kyriazi P, Headley DB, Pare D (2018). Multi-dimensional coding by basolateral amygdala neurons. Neuron.

[19] Adhikari A (2015). Basomedial amygdala mediates top-down control of anxiety and fear. Nature.

[20] Cho J-H, Deisseroth K, Bolshakov VY (2013). Synaptic encoding of fear extinction in mPFC-amygdala circuits. Neuron.

[21] Loewke AC, Minerva AR, Nelson AB, Kreitzer AC, Gunaydin LA (2021). Frontostriatal projections regulate innate avoidance behavior. J. Neurosci..

[22] Friedman A (2015). A corticostriatal path targeting striosomes controls decision-making under conflict. Cell.

[23] Balleine BW, O’Doherty JP (2010). Human and rodent homologies in action control: corticostriatal determinants of goal-directed and habitual action. Neuropsychopharmacology.

[24] Gremel CM, Costa RM (2013). Orbitofrontal and striatal circuits dynamically encode the shift between goal-directed and habitual actions. Nat. Commun..

[25] Hart G, Bradfield LA, Balleine BW (2018). Prefrontal corticostriatal disconnection blocks the acquisition of goal-directed action. J. Neurosci..

[26] Pitts EG, Li DC, Gourley SL (2018). Bidirectional coordination of actions and habits by TrkB in mice. Sci. Rep..

[27] Hart G, Bradfield LA, Fok SY, Chieng B, Balleine BW (2018). The bilateral prefronto-striatal pathway is necessary for learning new goal-directed actions. Curr. Biol..

[28] Lerner TN (2015). Intact-brain analyses reveal distinct information carried by SNc dopamine subcircuits. Cell.

[29] Pennington ZT (2019). ezTrack: an open-source video analysis pipeline for the investigation of animal behavior. Sci. Rep..

[30] Moscarello JM, LeDoux JE (2013). Active avoidance learning requires prefrontal suppression of amygdala-mediated defensive reactions. J. Neurosci..

[31] Chen T-W (2013). Ultra-sensitive fluorescent proteins for imaging neuronal activity. Published: Nature.

[32] Burgos-Robles A, Vidal-Gonzalez I, Quirk GJ (2009). Sustained conditioned responses in prelimbic prefrontal neurons are correlated with fear expression and extinction failure. J. Neurosci..

[33] Likhtik E, Stujenske JM, Topiwala MA, Harris AZ, Gordon JA (2014). Prefrontal entrainment of amygdala activity signals safety in learned fear and innate anxiety. Nat. Neurosci..

[34] Dejean C (2016). Prefrontal neuronal assemblies temporally control fear behaviour. Nature.

[35] Do-Monte FH, Quiñones-Laracuente K, Quirk GJ (2015). A temporal shift in the circuits mediating retrieval of fear memory. Nature.

[36] Rozeske RR (2018). Prefrontal-periaqueductal gray-projecting neurons mediate context fear discrimination. Neuron.

[37] Kravitz AV, Kreitzer AC (2012). Striatal mechanisms underlying movement, reinforcement, and punishment. Physiology.

[38] Yu, K., Garcia da Silva, P., Albeanu, D. F. & Li, B. Central amygdala somatostatin neurons gate passive and active defensive behaviors. *J. Neurosci.* **36**, 6488–6496 (2016). 10.1523/JNEUROSCI.4419-15.2016 PMC5015784 27307236

