# Supplementary material for: Divergent encoding of active avoidance behavior in corticostriatal and corticolimbic projections
Source: Sci Rep. 2022 Jun 24;12:10731. doi: 10.1038/s41598-022-14930-3 (PMC9232563; doi:10.1038/s41598-022-14930-3)
Supplement: Supplementary file 1 — Supplementary Information. [file 41598_2022_14930_MOESM1_ESM.docx]

**SUPPLEMENTARY METHODS**

**Stereotaxic Surgery**

Surgeries were done at 10-14 weeks of age. Mice were anesthetized using 5.0% isoflurane at an oxygen flow rate of 1 L/min and placed on top of a heating pad in a stereotaxic apparatus (Kopf Instruments). Anesthesia was maintained with 1.5-2.0% isoflurane for the duration of the surgery. Respiration and pinch response were monitored closely. Slow-release buprenorphine (.5mg/kg) and ketoprofen (1.6 mg/kg) were administered subcutaneously at the start of surgery. The incision area was shaved and cleaned with ethanol and betadine. Lidocaine (0.5%) was administered topically on the scalp. An incision was made along the midline and bregma was measured. Virus was injected (as described below) using a 10 µL nanofil syringe (World Precision Instruments) with a 33-gauge beveled needle. The needle was facing anterior for dmPFC injections and medial for DMS/BLA injections. We used an injection rate of 100 nL/min with a 10-minute delay before retracting the needle. Mice recovered in a clean cage on top of a heating pad and a subsequent injection of ketoprofen (1.6 mg/kg) was given the following day.

**Injection of virus for GcaMP expression (fiber photometry)**

*dmPFC pyramidal neurons:*

We expressed GcaMP6f in dmPFC pyramidal neurons using an adeno-associated virus (AAV) vector with serotype 5 to drive the expression of GcaMP6f-WPRE-SV40 under the CaMKII promoter in wild-type mice. Coordinates (in millimeters relative to bregma) for injection into the dmPFC were 1.8 A/P, -.35 M/L, -2.4 D/V. We injected 500 nL of virus and waited 4-5 weeks for expression before recording. For control animals we injected 500 nL of AAV5-CaMKIIa-eYFP (UNC Vector Core) into the dmPFC.

pENN.AAV.CamKII.GcaMP6f.WPRE.SV40 was a gift from James M. Wilson (Addgene viral prep # 100834-AAV5; http://n2t.net/addgene:100834 ; RRID:Addgene_100834). Titer 4.12E+13. AAV5-CaMKIIa-eYFP was a gift from Karl Deisseroth and packaged by the UNC Vector Core. Titer 3.60E+12.

*dmPFC-DMS and dmPFC-BLA projection neurons:*

We expressed GcaMP6m in dmPFC neurons projecting to either the DMS or BLA using a dual virus retrograde targeting strategy in wild-type mice. We used an adeno-associated virus (AAV) vector with serotype 1 to drive the expression of the Cre-dependent construct Flex-GcaMP6m-WPRE-SV40 under the synapsin (Syn) promoter (Addgene) in the dmPFC. Additionally, in the downstream target region (DMS or BLA) we injected a retrograde canine adenovirus type 2 (CAV2) to drive expression of Cre recombinase (Institut de Génétique Moléculaire de Montpellier, Montpellier, France) as well as an AAV8 virus to drive the expression of mCherry under the human synapsin (hSyn) promotor (UNC Vector Core; to visualize CAV2 injection location). Coordinates (in millimeters relative to bregma) for dmPFC injections were the same as above, coordinates for the DMS were .8 A/P, -1.5 M/L, -3.5 D/V, and coordinates for the BLA were -1.4 A/P, -3.3 M/L, -4.9 D/V. We injected 1500 nL of Syn-Flex-GcaMP6m in the dmPFC and either 350 nL of each CAV2-Cre and hSyn-mCherry in the DMS or 250 nL of each in the BLA. We waited 4-5 weeks for expression before recording. For control animals we injected 1500 nL AAV5-EF1a-DIO-eYFP-WPRE-hGH (Addgene) into the dmPFC.

pAAV.Syn.Flex.GcaMP6m.WPRE.SV40 was a gift from The Genetically Encoded Neuronal Indicator and Effector Project (GENIE) & Douglas Kim (Addgene viral prep # 100838-AAV1; http://n2t.net/addgene:100838 ; RRID:Addgene_100838). Titer 2.10E+13. pAAV-Ef1a-DIO EYFP was a gift from Karl Deisseroth (Addgene viral prep # 27056-AAV5 ; http://n2t.net/addgene:27056 ; RRID:Addgene_27056). Titer 2.40E+13. CAV2-Cre was packaged by the Plateforme de Vectorologie de Montpellier. Titer 1.00E+13. AAV8-hSyn-mCherry was a gift from Karl Deisseroth and packaged by the UNC Vector Core. Titer 4.1E +12.

**Active Avoidance Behavior**

Each active avoidance trial consisted of a 10 second light cue followed by 10 seconds of light plus 0.3 mA shock. Light and shock were presented on the shock floor the mouse was currently on at the initiation of the trial. Mice were able to avoid the shock altogether by moving onto the other unlit shock floor during the 10 second light only period. This was considered a successful active avoidance trial. Trials in which the mouse failed to move to the other unlit shock floor during the 10 seconds of light only are considered unsuccessful trials. Training continued until the group average was at or above 80% successful avoidance (24 out of 30 trials). Location of the mice was recorded and quantified using Ethovision XT software.

**Fiber Photometry Recording**

*In vivo* calcium data were acquired using a custom-built rig based on a previously described setup (Lerner et al., 2015). This setup was controlled by an RZ5P fiber photometry processor (TDT, Alachua, FL, USA) and Synapse software (TDT). The RZ5P/Synapse software controlled a 4 channel LED Driver (DC4100, Thorlabs, Newton, NJ, USA) which in turn controlled two fiber-coupled LEDS: 470 nm for GCaMP stimulation and 405 nm to control for artifactual fluorescence (M470F3, M405FP1, Thorlabs). These LEDs were sinusoidally modulated at 210 Hz (470 nm) and 320 Hz (405 nm) and connected to a Fluorescence Mini Cube with 4 ports (Doric Lenses) and the combined LEF output was connected through a fiber optic patch cord (0.48 NA, 400 µm, Doric Lenses) to the cannula via a ceramic sleeve (Thorlabs). The emitted light was focused onto a Visible Femtowatt Photoreceiver Module (Model 2151, Newport, AC low) and sampled at 60 Hz. Video tracking software (Ethovision, Noldus) was synchronized to the photometry setup using TTL pulses generated every 10 seconds following the start of the Noldus trial. Raw photoreceiver data was extracted and analyzed using custom scripts in Matlab (The MathWorks, Natick, MA, USA). The two output signal data was demodulated from the raw signal based on the LED modulation frequency. To normalize the data and correct for bleaching, the 405 nm channel signal was fitted to a polynomial over time and subtracted from the 470 nm GCaMP signal, yielding the DF/F value.

**Fiber Photometry Data Analysis**

Quantification was done using the average signal across the following time windows:

CS onset: Baseline (-1 to 0 sec), CS response (0 to 1 sec)

CS successful vs. unsuccessful: Baseline (-1 to 0 sec), Initial CS response (0 to 1 sec), Pre-avoidance (1 to 2 sec), Post-avoidance (9 to 10 sec)

Avoidance movement: Baseline (-10 to -7 sec), Pre-avoidance (-3 to -0 sec), Avoidance (0 to 3 sec), Post-avoid: (7 to 10 sec)

ITI movement: Baseline (-10 to -8 sec), Pre-movement (-3 to -1 sec), Movement (-1 to 1 sec), Post-movement: (1 to 3 sec)

Freezing: Baseline (-2 to -1.5 sec), Freezing (0 to 0.5 sec)

**Perfusions and Histology**

Following the conclusion of behavioral experiments, animals were anesthetized using 5% isoflurane and given a lethal dose (1.0 mL) cocktail of ketamine/xylazine (10 mg/ml ketamine, 1 mg/ml xylazine). They were then transcardially perfused with 10 mL of 1X PBS followed by 10 mL 4% paraformaldehyde (PFA). Brains were extracted and left in 4% PFA overnight and then transferred to a 30% sucrose solution until slicing. The brains were frozen and sliced on a sliding microtome (Leica Biosystems, Wetzlar, Germany) and placed in cryoprotectant in a well-plate. Slices were then washed in 1X PBS, mounted on slides (Fisherbrand Superfrost Plus, ThermoFisher Scientific, Waltham, MA, USA) and air dried (covered). ProLong Gold antifade reagent (Invitrogen, ThermoFisher Scientific)  was injected on top of the slices and a cover slip (Slip-rite, ThermoFisher Scientific) was placed on top and the slides were left to dry overnight (covered). Viral injection, fiber photometry cannula implant, and optogenetic cannula implant placements were histologically verified on a fluorescence microscope (Leitz DMRB, Leica).

**SUPPLEMENTARY FIGURES**

**
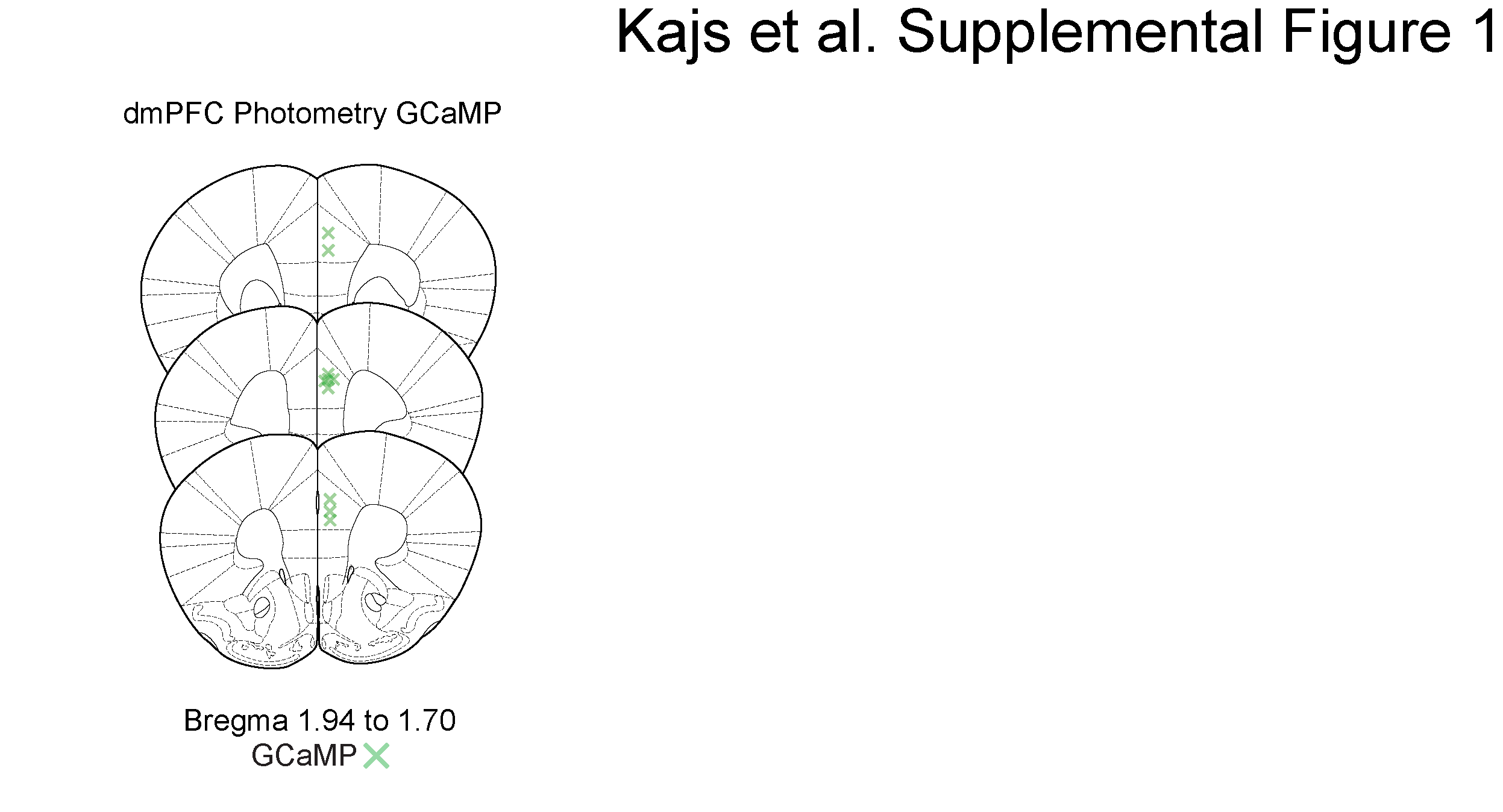
**

**Supplemental Figure 1.** Targeting for dmPFC photometry surgeries. Verification of GCaMP virus injection in dmPFC (N = 10 mice). Injections corresponded mostly to the prelimbic region.


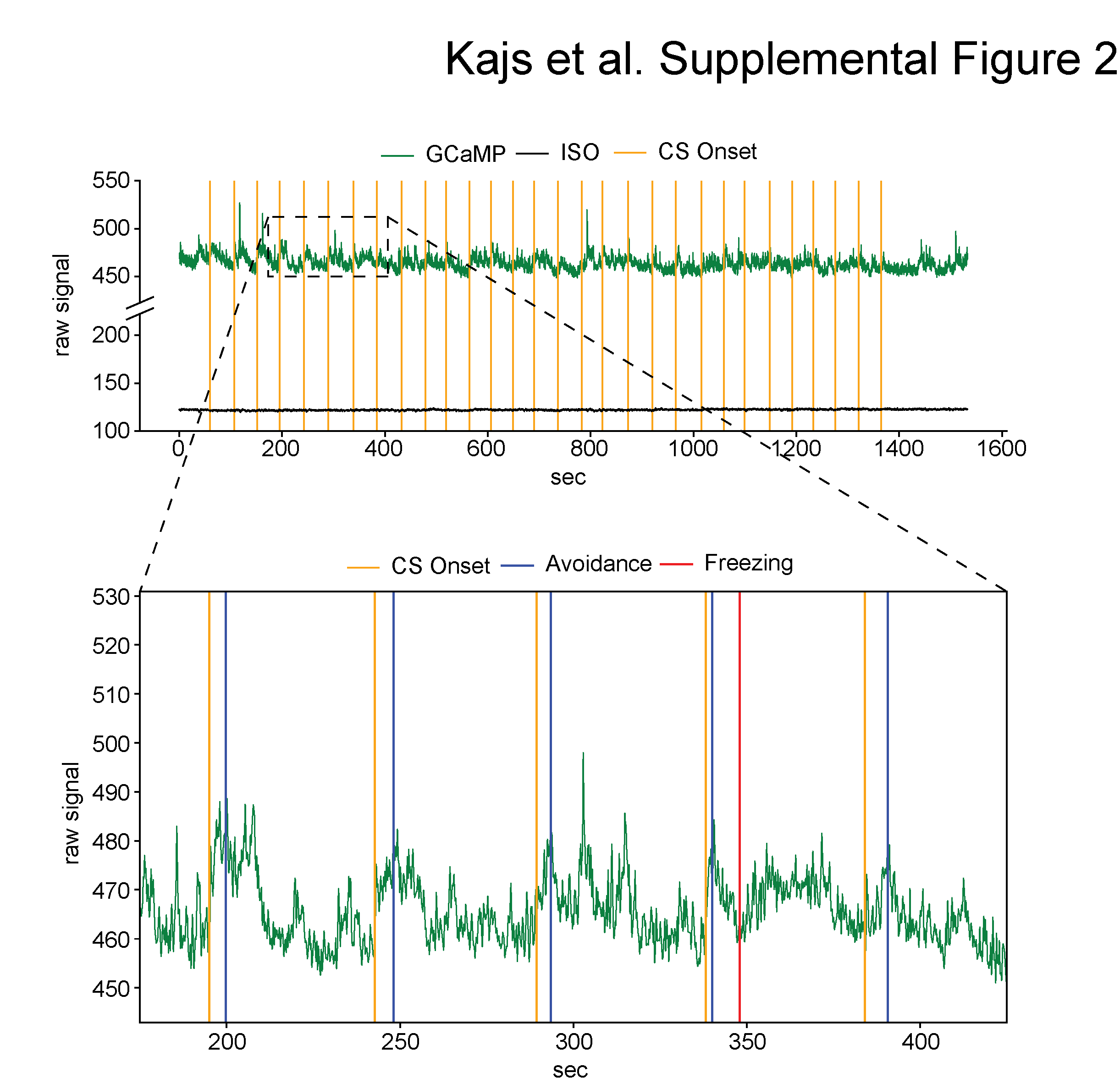


**Supplemental Figure 2.** Example raw dmPFC GCAMP trace with labelled events. Raw trace shows increase in calcium signal during CS onset (orange lines) as well as avoidance onset (blue lines) and decreases in calcium signal during freezing onset (red lines).


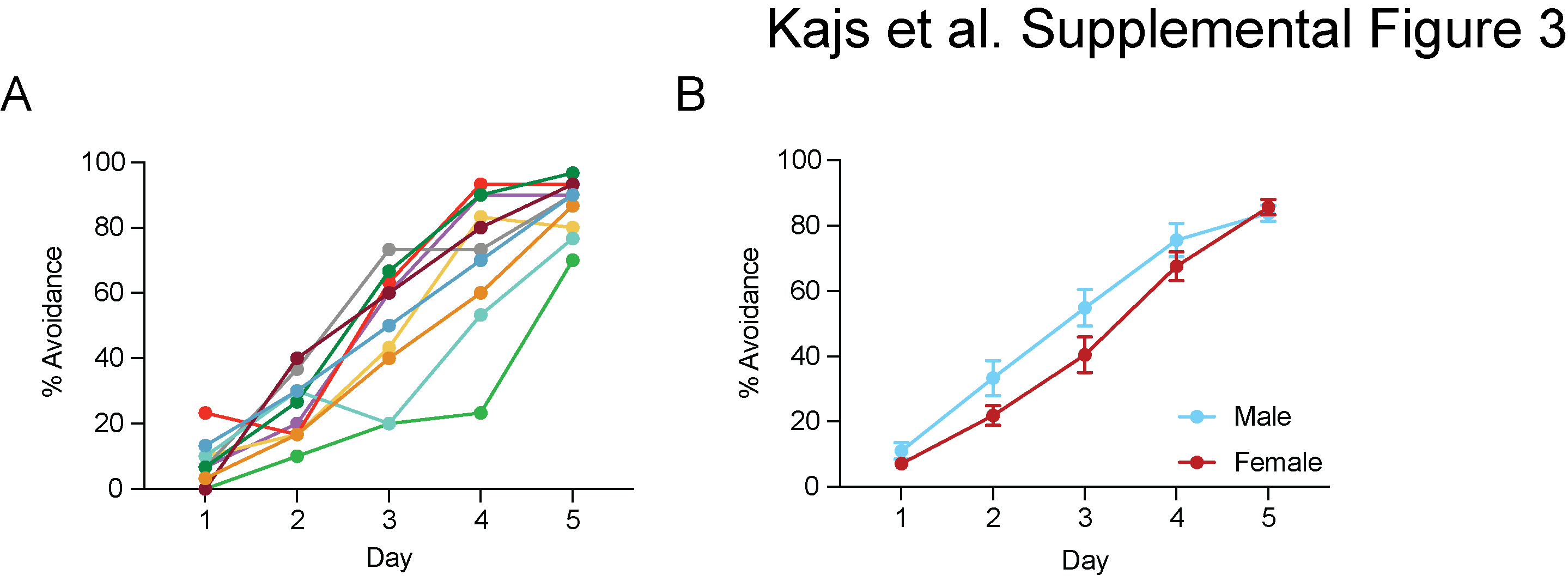


**Supplemental Figure 3.** Learning curves for the dmPFC cohort by individual animal and by sex. (A) Learning curves for individual animals (n = 10 mice). (B) Learning curves for male and female mice did not significantly differ from each other throughout learning. Data comes from animals from all cohorts (dmPFC, dmPFC-DMS, and dmPFC-BLA cohorts).

 
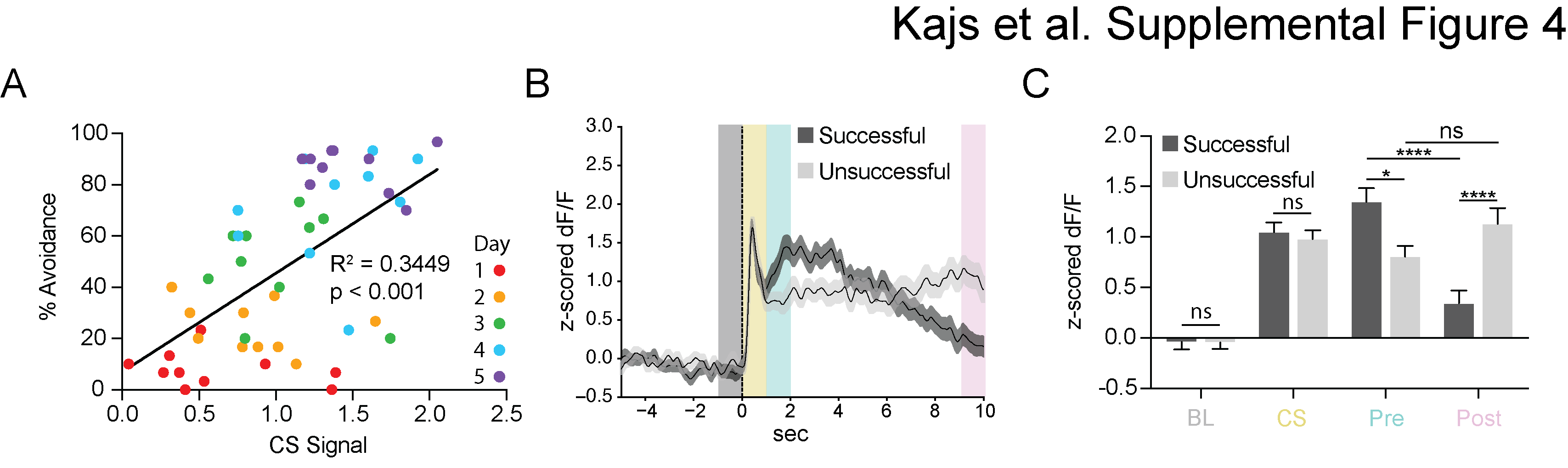


**Supplemental Figure 4.** dmPFC neural activity at CS onset correlates with percent avoidance and does not differ between successful versus unsuccessful trials. (A) Significant positive correlation between individual animal dmPFC calcium signal during the first second of CS onset and percent successful avoidance from the same recording day. Data points (n = 50) are from individual animals (n = 10) during day 1-5 of learning. (Linear Regression, slope = 38.52, y intercept = 6.937, R^2^ = 0.3449, F = 25.28, p < 0.001) (B) PETH of calcium signal in dmPFC aligned to CS onset for successful (dark grey line) and unsuccessful (light grey line) trials shows differences in later parts of the trace when avoidances normally do or do not occur. Trials from Day 3 were used since equal numbers of successful and unsuccessful trials occur on this training day. Grey box, baseline period (BL); yellow box, CS response period (CS); teal box, pre avoidance period (Pre); pink box, post avoidance period (Post) (B) Quantification of the CS onset PETH shows no differences in calcium signal between successful and unsuccessful trials during the baseline period (-1 to 0 s) and the CS response period (0 to 1 s). However, the calcium signal in the dmPFC is significantly increased during successful trials compared to unsuccessful trials during the pre avoidance period (1 to 2 s) and significantly decreased during successful trials compared to unsuccessful trials during the post avoidance period (9 to 10 s) (Two-way ANOVA, Task Period x Trial Type p < 0.0001, Task Period p < 0.0001, Trial Type p = 0.5807; Sidak’s Multiple Comparisons Test, Successful Baseline vs Unsuccessful Baseline p > 0.9999, Successful CS Response vs Unsuccessful CS Response p = 0.986, Successful Pre Avoidance vs Unsuccessful Pre Avoidance p = 0.0022, Successful Post Avoidance vs Unsuccessful Post Avoidance p < 0.0001; N = 10 mice, Successful n = 147 trials, Unsuccessful n = 153 trials). ns = not significant, * p < 0.0332, **** p < 0.0001.

**
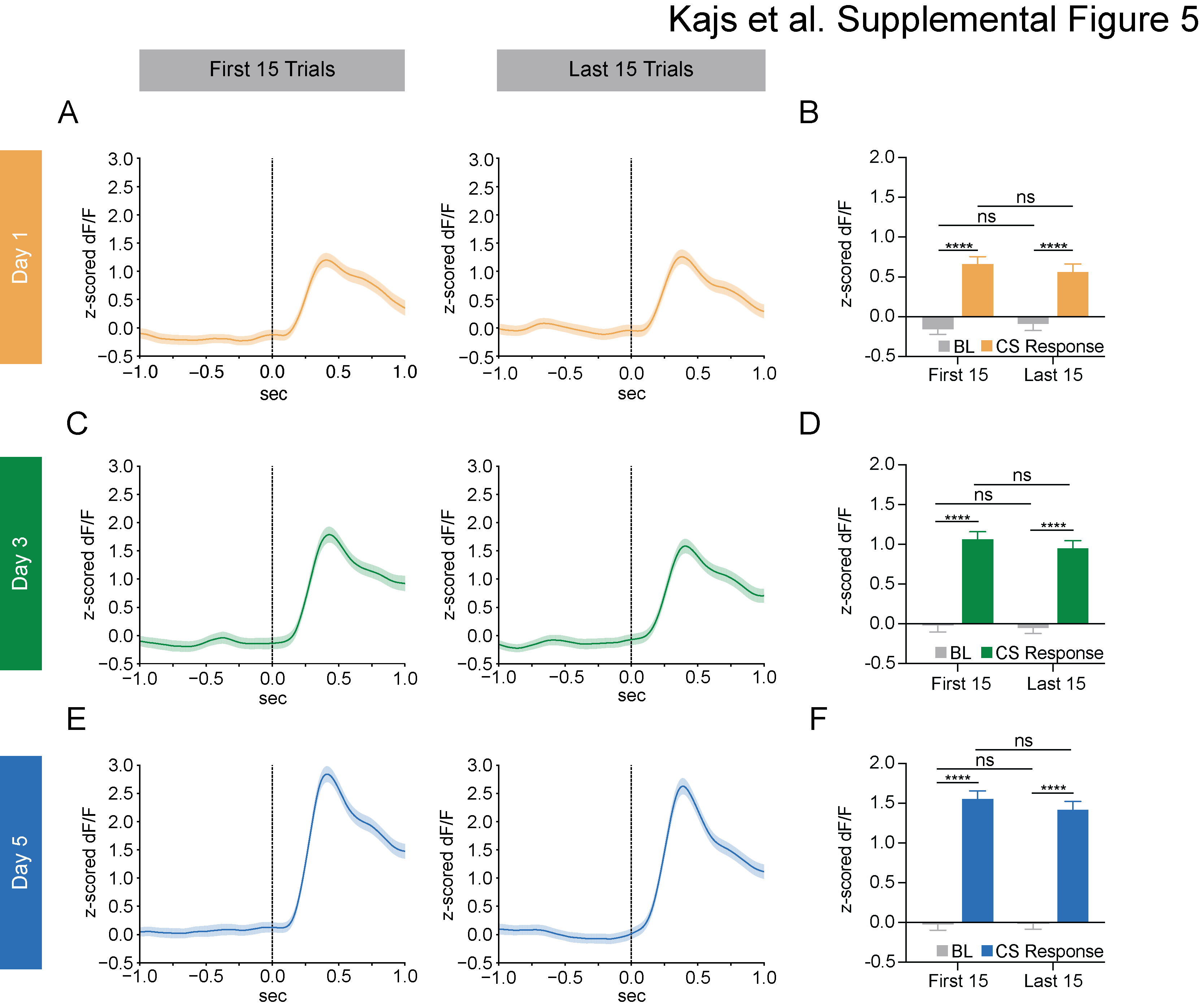
**

**Supplemental Figure 5.** No within-day differences in dmPFC neural activity at CS onset. (A) PETHs of dmPFC calcium signal show no differences between the first 15 trials (left) and the last 15 trials (right) on day 1 of training. (B) Quantification of the day 1 PETHs show no significant differences in calcium signal between the first 15 trials and the last 15 trials during the baseline (-1 to 0 s) or CS (0 to 1 s) periods (Two-way ANOVA, Part of Session x Task Period p = 0.3176, Part of Session p = 0.8385, Task Period p < 0.0001; Sidak’s Multiple Comparisons Test, First 15 Baseline vs Last 15 Baseline p = 0.994, First 15 Baseline vs First 15 CS p < 0.0001, First 15 CS vs Last 15 CS p = 0.9509, Last 15 Baseline vs Last 15 CS p < 0.0001; N = 10 mice, First 15 n = 150 trials, Last 15 n = 150 trials). (C) PETHs of dmPFC calcium signal show no differences between the first 15 trials (left) and the last 15 trials (right) on day 3 of training. (D) Quantification of the day 3 PETHs show no significant differences in calcium signal between the first 15 trials and the last 15 trials during the baseline (-1 to 0 s) or CS (0 to 1 s) periods (Two-way ANOVA, Part of Session x Task Period p = 0.6153, Part of Session p = 0.3854, Task Period p < 0.0001; Sidak’s Multiple Comparisons Test, First 15 Baseline vs Last 15 Baseline p > 0.9999, First 15 Baseline vs First 15 CS p < 0.0001, First 15 CS vs Last 15 CS p = 0.9116, Last 15 Baseline vs Last 15 CS p < 0.0001; N = 10 mice, First 15 n = 150 trials, Last 15 n = 150 trials). (E) PETHs of dmPFC calcium signal show no differences between the first 15 trials (left) and the last 15 trials (right) on day 5 of training. (F) Quantification of the day 5 PETHs show no significant differences in calcium signal between the first 15 trials and the last 15 trials during the baseline (-1 to 0 s) or CS (0 to 1 s) periods (Two-way ANOVA, Part of Session x Task Period p = 0.388, Part of Session p = 0.4610, Task Period p < 0.0001; Sidak’s Multiple Comparisons Test, First 15 Baseline vs Last 15 Baseline p > 0.9999, First 15 Baseline vs First 15 CS p < 0.0001, First 15 CS vs Last 15 CS p = 0.8329, Last 15 Baseline vs Last 15 CS p < 0.0001; N = 10 mice, First 15 n = 150 trials, Last 15 n = 150 trials). ns = not significant, **** p < 0.0001.


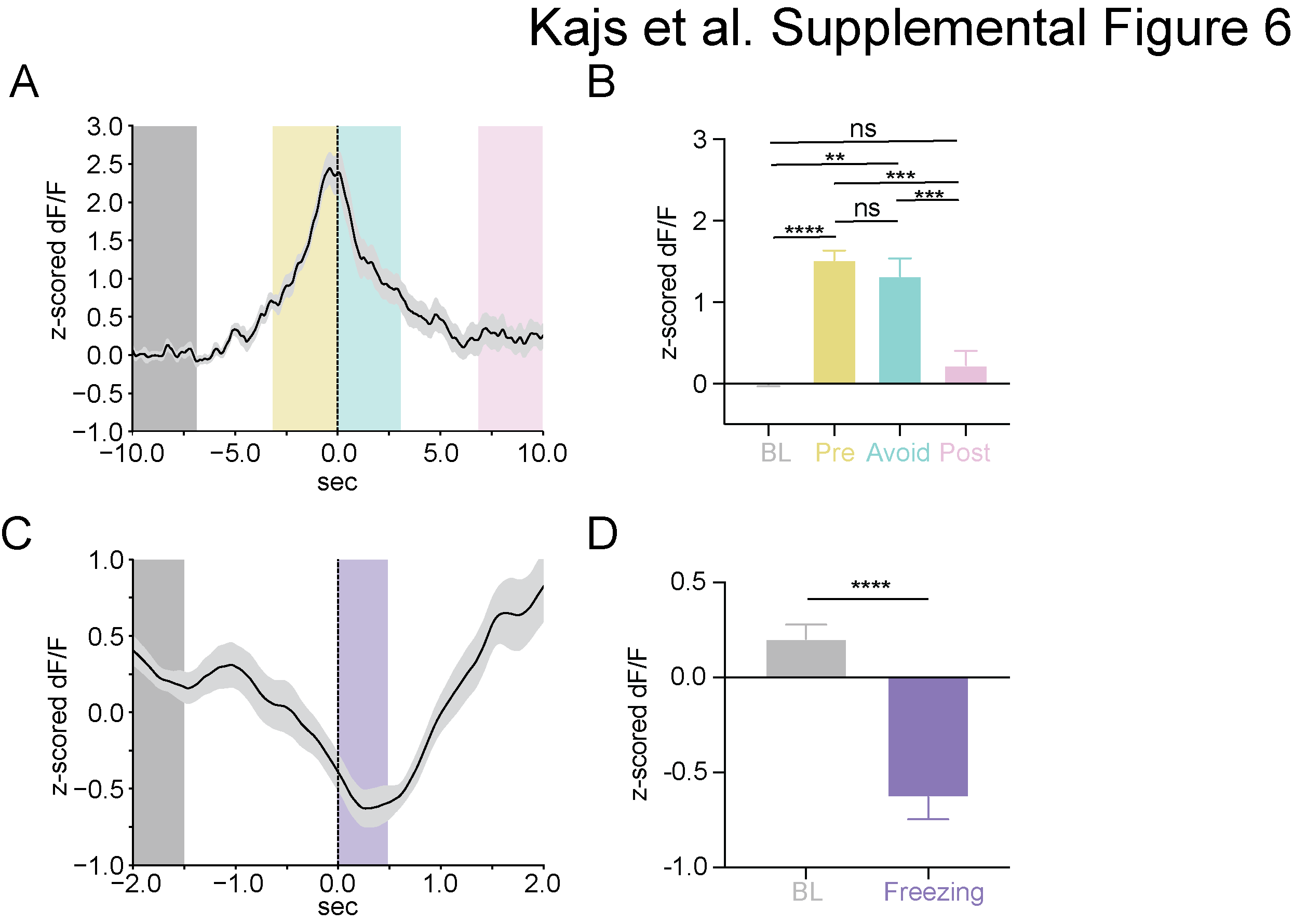


**Supplemental Figure 6.** dmPFC calcium signal during avoidance and freezing quantified by individual animal rather than behavioral events. Data were quantified using one average trace per animal as compared to averaging all individual traces from all individual behavioral events. This method allows each individual animal’s data to be weighed equally in the final quantification. (A) PETH shows an increase in calcium signal at avoidance onset on Day 5. Line with shading represents mean ± SEM. Grey box, baseline period (BL); yellow box, pre avoidance period (Pre); teal box, avoidance period (Avoid); pink box, post avoidance period (Post). (B) Quantification of avoidance PETH reveals significantly increased calcium signal in the pre avoid (-3 to 0 s) and avoid (0 to 3 s) periods but not the post avoid period (7 to 10 s) compared to the baseline period (-10 to -7 s) (Repeated Measures One-Way ANOVA F_(2.048, 18.43)_ = 40.09, p < 0.0001; Tukey’s Multiple Comparisons Test, Baseline vs Pre Avoid p < 0.0001, Baseline vs Avoid p = 0.0011, Baseline vs Post Avoid p = 0.5804, Pre Avoid vs Avoid p = 0.6887, Pre Avoid vs Post Avoid p = 0.00, Avoid vs Post Avoid p = 0.0001; N = 10 mice, n = 50 (10 mice x 5 days)). (C) PETH shows decrease in calcium signal at freezing onset on Day 1. Line with shading represents mean ± SEM. Grey box, baseline period (BL); Purple box, freezing period (Freezing). (D) Quantification of freezing PETH shows significant decrease in calcium signal during the freezing period (0-0.5 s) compared to the baseline period (-2 to -1.5 s) (Paired t-test t = 7.605, df = 9, p < 0.0001; N = 10 mice, n = 50 (10 mice x 5 days)). ns = not significant, ** p < 0.0021, *** p < 0.0002, **** p < 0.0001.


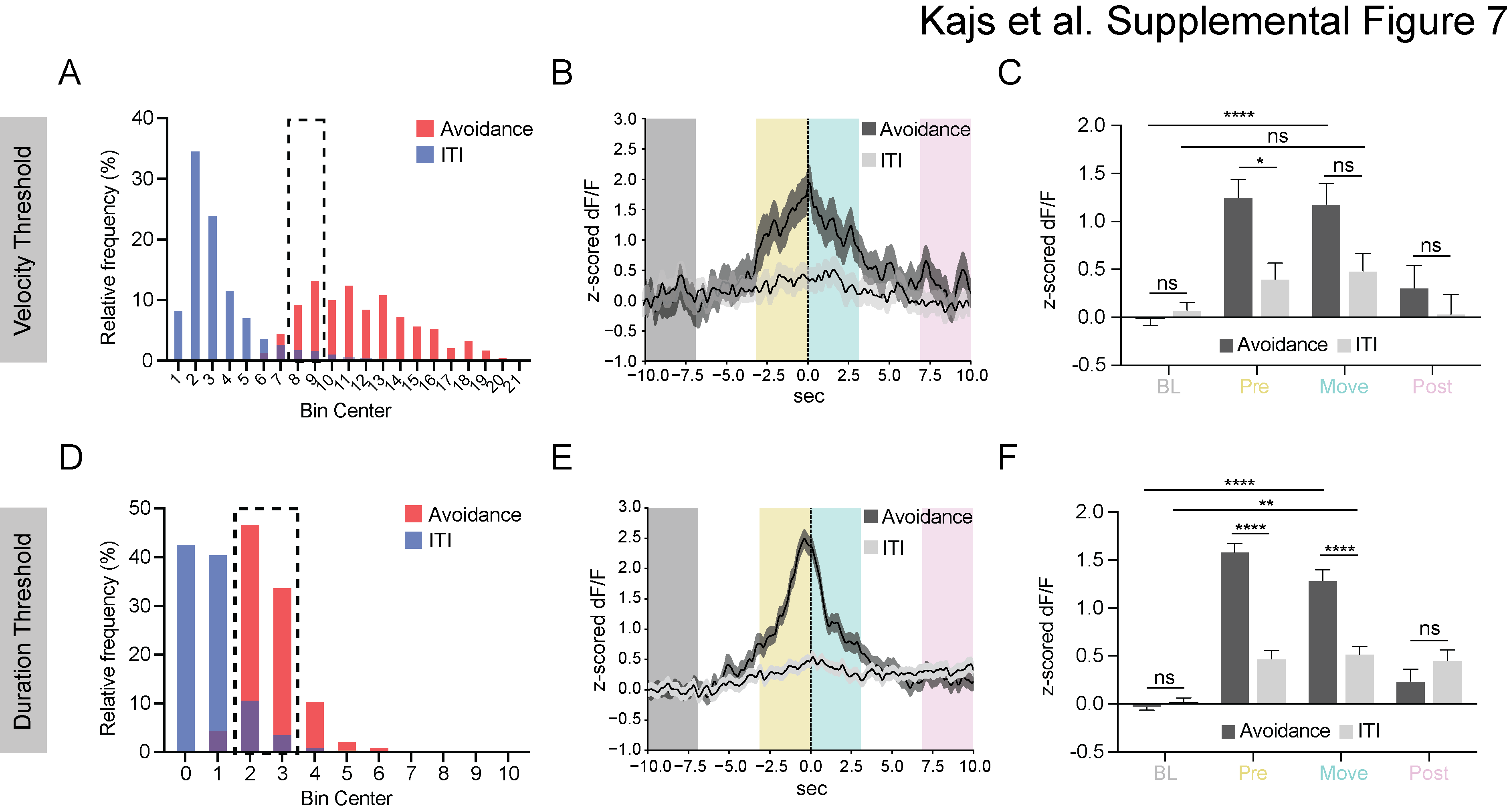


**Supplemental Figure 7.** Increased activity in the dmPFC during avoidance is not purely movement-related. (A) Distribution of movement velocities for intertrial (ITI) (blue) and avoidance (red) movements and their overlap (purple). (B) PETH of ITI and avoidance movements of similar velocities (7.5 cm/s to 9.5 cm/s) aligned to movement onset shows increase in calcium signal during avoidance movements that is not seen during ITI movements. Grey box, baseline period (BL); yellow box, pre movement period (Pre); teal box, movement period (Move); pink box, post movement period (Post) (C) Quantification of similar velocity movement PETH shows dmPFC calcium signal is significantly increased during avoidance movements compared to ITI movements during the pre-movement period (-3 to 0 s), but not during baseline (-10 to -7 s), movement (0 to 3 s), or post-movement (7 to 10 s) periods (Two-way ANOVA, Task Period x Movement Type F_(3, 464)_ = 2.926, p = 0.0335, Task Period p < 0.0001, Movement Type p = 0.0006; Sidak’s Multiple Comparisons Test, Avoidance Baseline vs ITI Baseline p > 0.9999, Avoidance Baseline vs Avoidance Movement p < 0.0001, ITI Baseline vs ITI Movement p = 0.9428, Avoidance Pre-Movement vs ITI Pre-Movement p = 0.0202, Avoidance Movement vs ITI Movement p = 0.1417, Avoidance Post-Movement vs ITI Post-Movement p = 0.9999; N = 10 mice, Avoidance n = 58 trials, ITI n = 60 trials). (D) Distribution of movement durations for ITI (blue) and avoidance (red) movements and their overlap (purple). (E) PETH of ITI and avoidance movements of similar durations (1.5 s to 3.5 s) aligned to movement onset shows sharp increase in calcium signal during avoidance movements that is not seen during ITI movements. (F) Quantification of similar movement duration PETH shows dmPFC calcium signal is significantly increased during avoidance movements compared to ITI movements during pre-movement (-3 to 0 s) and movement (0 to 3 s) periods but not during the baseline (-10 to -7 s) or post-movement (7 to 10 s) periods (Two-way ANOVA, Task Period x Movement Type F_(3,1720)_ = 24.62, p < 0.0001, Task Period p < 0.0001, Movement Type p < 0.0001; Sidak’s Multiple Comparisons Test, Avoidance Baseline vs ITI Baseline p > 0.9999, Avoidance Baseline vs Avoidance Movement p < 0.0001, ITI Baseline vs ITI Movement p = 0.0020, Avoidance Pre-Movement vs ITI Pre-Movement p < 0.0001, Avoidance Movement vs ITI Movement p < 0.0001, Avoidance Post-Movement vs ITI Post-Movement p = 0.9345; N = 10 mice, Avoidance n = 205 trials, ITI n = 227 trials).  ns = not significant, * p < 0.0332, ** p < 0.0021, **** p < 0.0001.


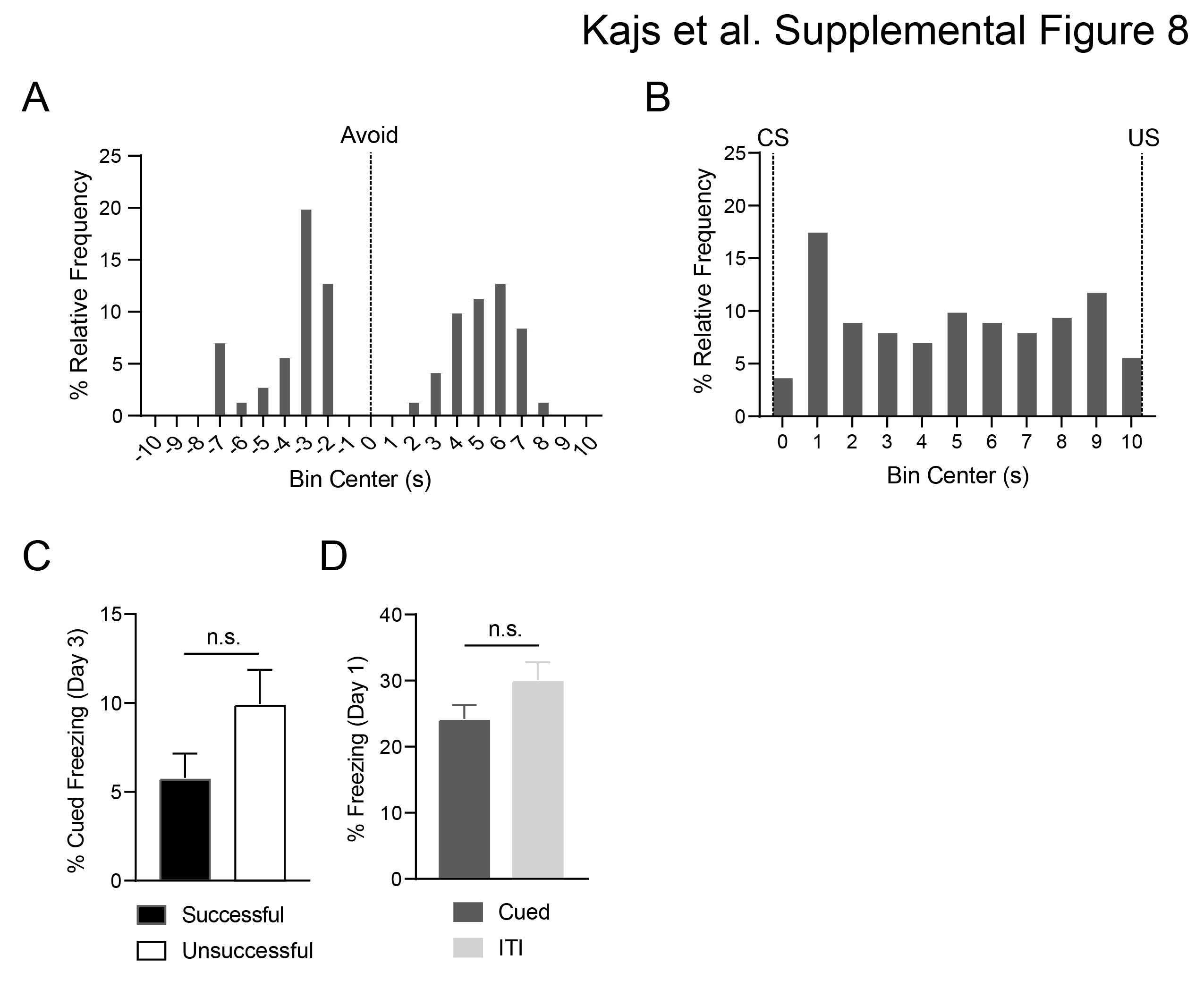


**Supplemental Figure 8.** Additional analyses of freezing behavior for the dmPFC cohort. (A) Histogram of percent relative frequency of cued freezing on Day 3 before (-10 to 0 s) and after (0 to 10 s) avoidance onset (0 s) shows that cued freezing occurs evenly both before and after avoidance onset. (B) Histogram of percent relative frequency of cued freezing on Day 3 between CS onset (0 s) and US onset (10 s) shows a fairly uniform distribution of freezing across the CS-only period. (C) There was no significant difference in cued freezing on Day 3 between successful and unsuccessful trials (Paired t-test t = 1.480, df = 9, p = 0.1729; N = 10 mice). (D) There was no significant difference in percent freezing during the cue (cued freezing) and percent freezing during the intertrial interval period (ITI freezing) on Day 1 (Paired t-test t = 1.957, df = 9, p = 0.0821; N = 10 mice).

**
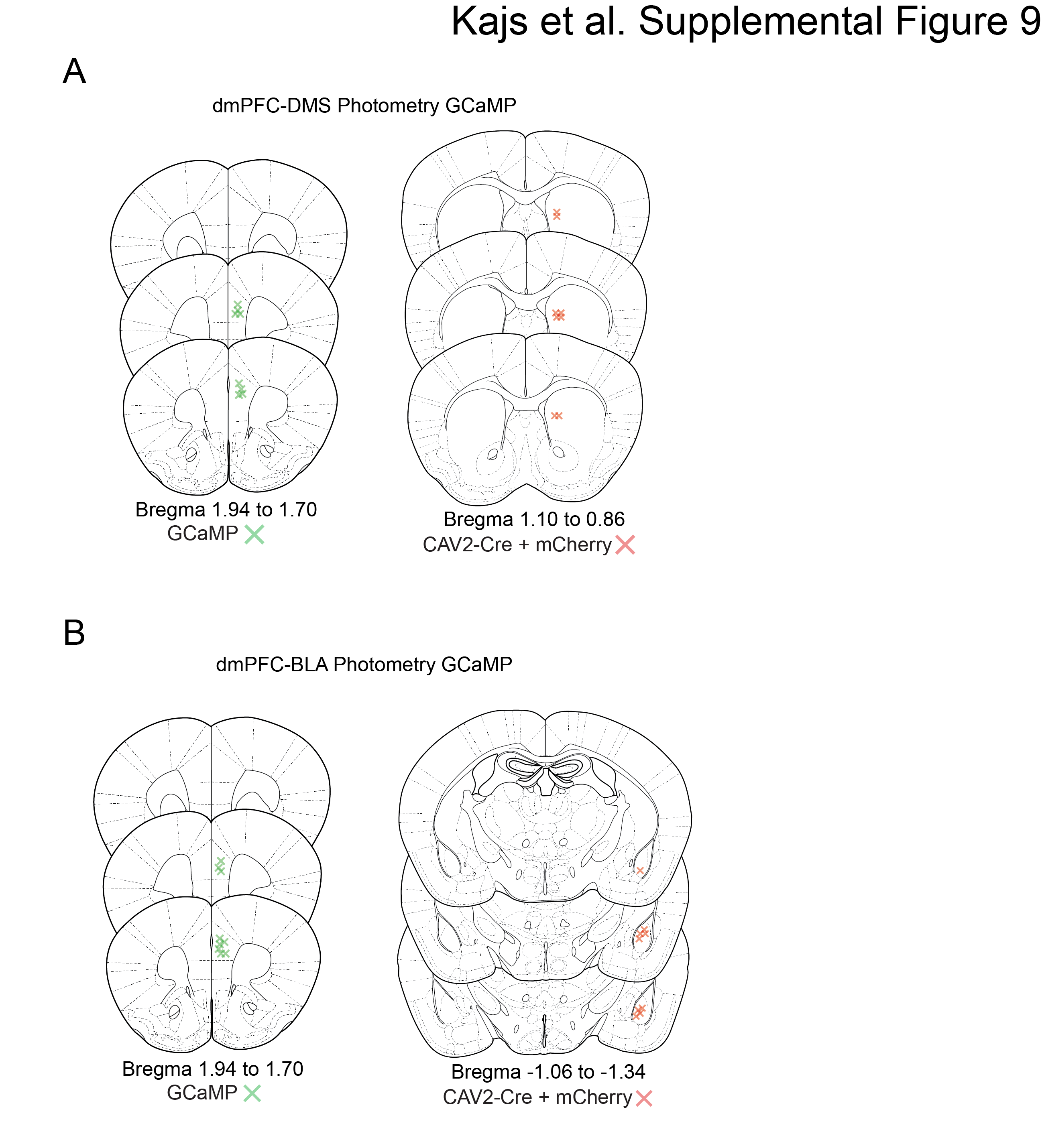
**

**Supplemental Figure 9.** Targeting for dmPFC-DMS and dmPFC-BLA photometry surgeries. (A) Verification of GCaMP virus injection in dmPFC (left) and CAV2-Cre + mCherry viral injection in DMS (right) for dmPFC-DMS cohort (N = 8 mice). (B) Verification of GCaMP virus injection in dmPFC (left) and CAV2-Cre + mCherry viral injection in BLA (right) for dmPFC-BLA cohort (N = 9 mice).


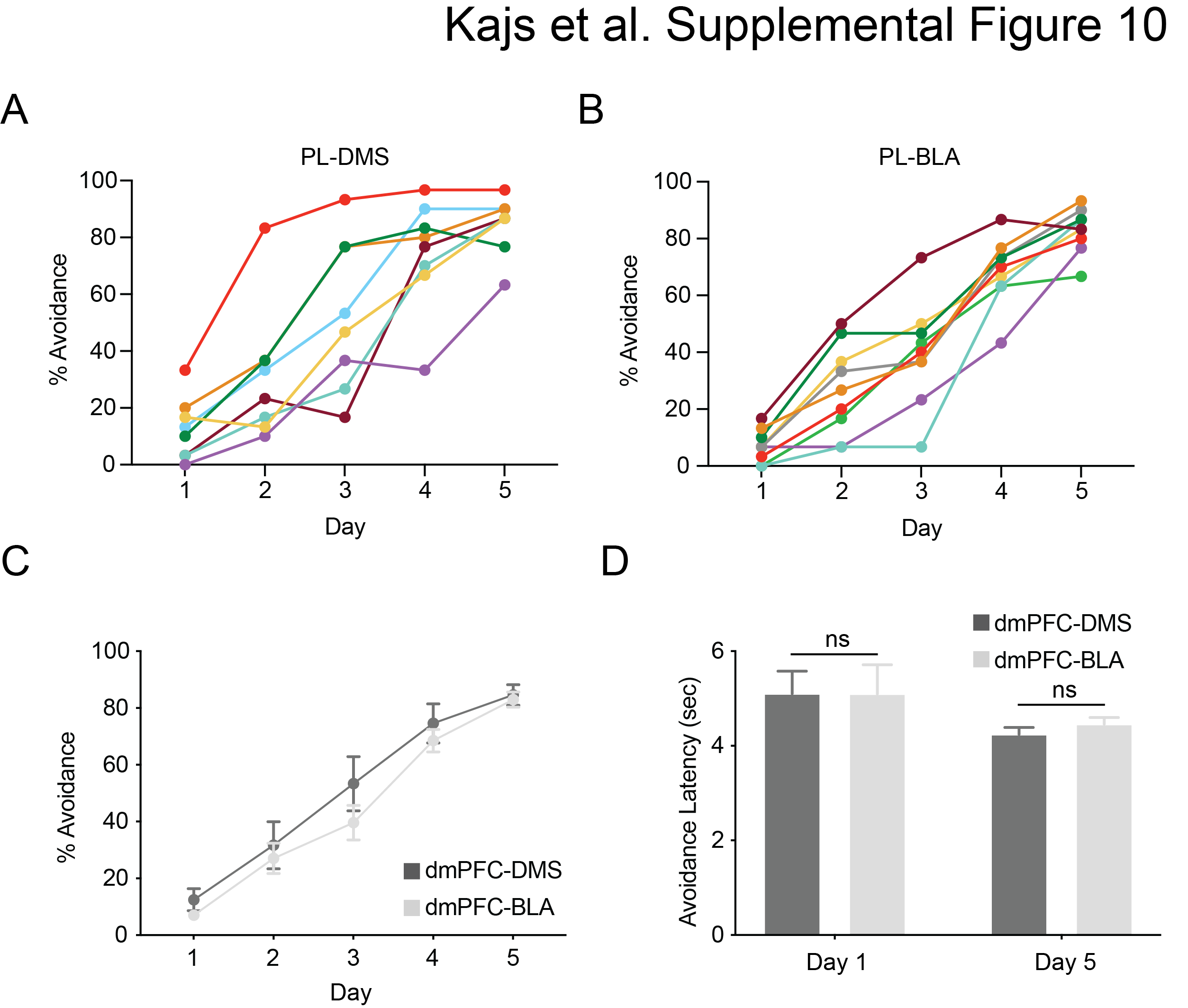


**Supplemental Figure 10.** Extended behavioral data for dmPFC-DMS and dmPFC-BLA cohorts. (A) Learning curves for individual animals from the dmPFC-DMS cohort (n = 8 mice). (B) Learning curves for individual animals from the dmPFC-BLA cohort (n = 9 mice). (C) dmPFC-DMS and dmPFC-BLA learning curves (all mice included) did not significantly differ from each other throughout learning. (D) Average avoidance latencies from the dmPFC-DMS and dmPFC-BLA cohorts did not significantly differ from each other during day 1 and day 5 of learning. ns = not significant.


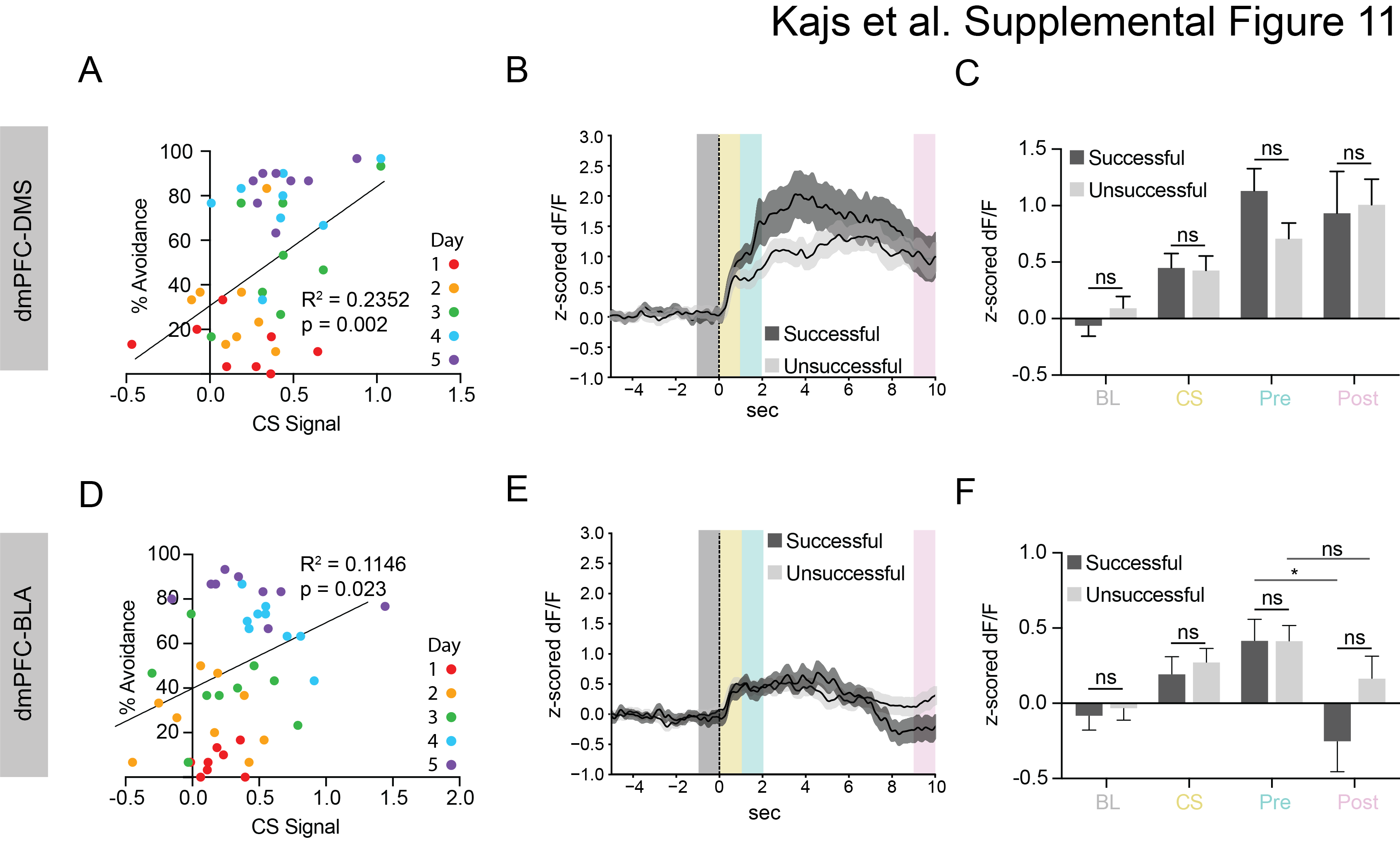


**Supplemental Figure 11.** dmPFC-DMS and dmPFC-BLA neural activity at CS onset correlates with percent avoidance and does not differ between successful versus unsuccessful trials. (A) Significant positive correlation between individual animal dmPFC-DMS calcium signal during the first second of CS onset and percent successful avoidance from the same recording day. Data points (n = 40) are from individual animals (n = 8) during day 1-5 of learning (Linear Regression, slope = 53.50, y-intercept = 33.63, R^2^ = 0.2352, F = 11.68, p = 0.002). (B) PETH of calcium signal in the dmPFC-DMS projection aligned to CS onset for successful (dark grey line) and unsuccessful (light grey line) trials shows no differences between successful and unsuccessful traces. Trials from Day 3 were used since equal numbers of successful and unsuccessful trials occur on this training day. Grey box, baseline period (BL); yellow box, CS response period (CS); teal box, pre avoidance period (Pre); pink box, post avoidance period (Post) (C) Quantification of the CS onset PETH shows no differences in calcium signal between successful and unsuccessful trials during the baseline period (-1 to 0 s), CS response period (0 to 1 s), pre avoidance period (1 to 2 s), or post avoidance period (9 to 10 s) for the dmPFC-DMS projection (Two-way ANOVA, Task Period x Trial Type p < 0.4554, Task Period p < 0.0001, Trial Type p = 0.7025; Sidak’s Multiple Comparisons Test, Successful Baseline vs Unsuccessful Baseline p = 0.9633, Successful CS Response vs Unsuccessful CS Response p > 0.9999, Successful Pre Avoidance vs Unsuccessful Pre Avoidance p = 0.4172, Successful Post Avoidance vs Unsuccessful Post Avoidance p = 0.9978; N = 8 mice, Successful n = 126 trials, Unsuccessful n = 114 trials). (D) Significant positive correlation between individual animal dmPFC-BLA calcium signal during the first second of CS onset and percent successful avoidance from the same recording day. Data points (n = 45) are from individual animals (n = 9) during day 1-5 of learning (Linear Regression, slope = 29.53, y-intercept = 36.03, R^2^ = 0.1146, F = 5.564, p = 0.023). (E) PETH of calcium signal in the dmPFC-BLA projection aligned to CS onset for successful (dark grey line) and unsuccessful (light grey line) trials shows no differences between successful and unsuccessful traces on Day 3. (F) Quantification of the CS onset PETH shows no differences in calcium signal between successful and unsuccessful trials during the baseline period (-1 to 0 s), the CS response period (0 to 1 s), the pre avoidance period (1 to 2 s), or the post avoidance period (9 to 10 s) for the dmPFC-BLA projection (Two-way ANOVA, Task Period x Trial Type p = 0.3127, Task Period p < 0.0001, Trial Type p = 0.1204; Sidak’s Multiple Comparisons Test, Successful Baseline vs Unsuccessful Baseline p > 0.9999, Successful CS Response vs Unsuccessful CS Response p > 0.9999, Successful Pre Avoidance vs Unsuccessful Pre Avoidance p > 0.9999, Successful Pre Avoidance vs Successful Post Avoidance p = 0.0137, Unsuccessful Pre Avoidance vs Unsuccessful Post Avoidance p = 0.9679, Successful Post Avoidance vs Unsuccessful Post Avoidance p = 0.3839; N = 9 mice, Successful n = 109 trials, Unsuccessful n = 161 trials). ns = not significant, * p < 0.05.

**
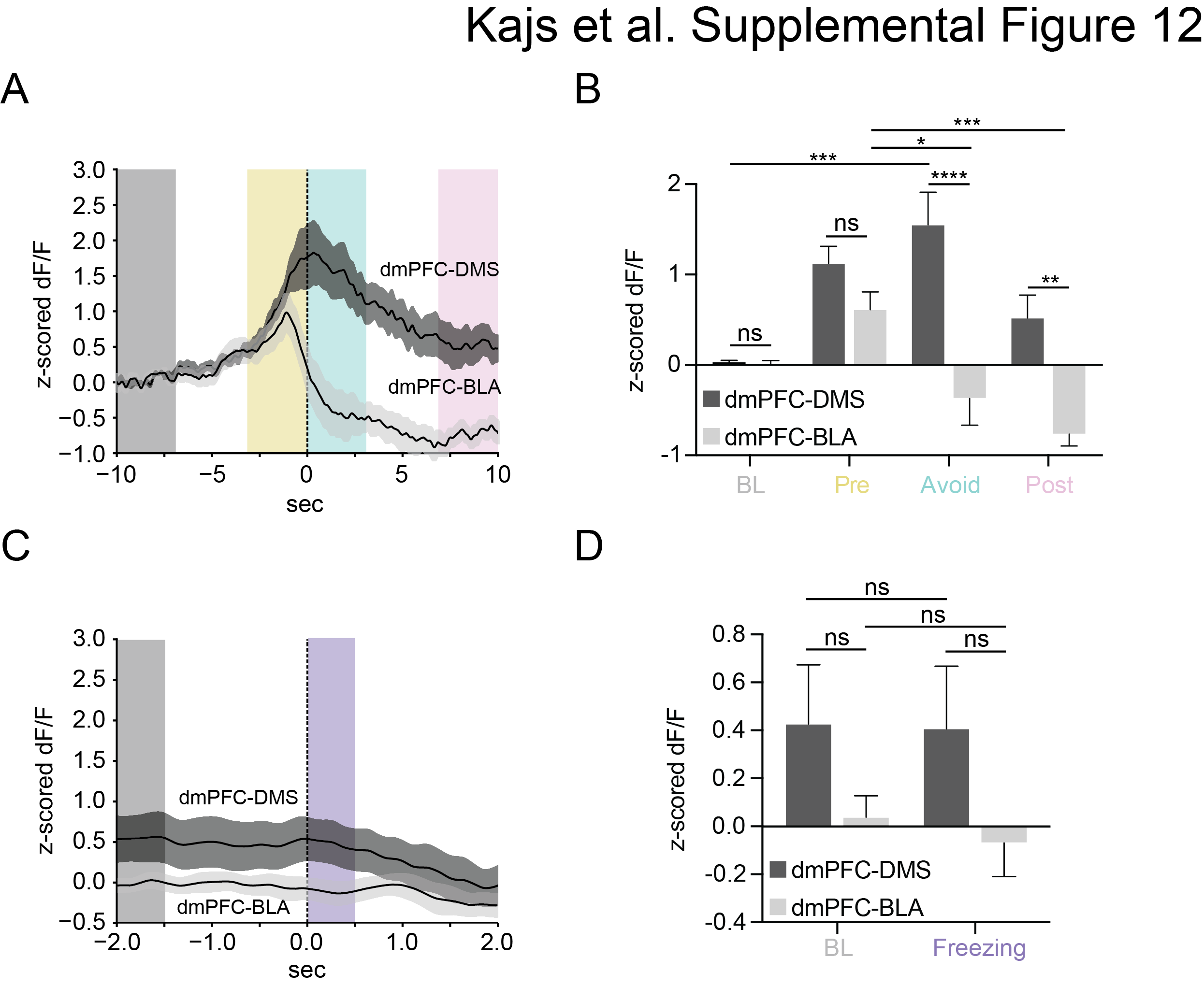
**

**Supplemental Figure 12.** dmPFC-DMS and dmPFC-BLA calcium signal during avoidance and freezing quantified by individual animal rather than behavioral events. Data were quantified using one average trace per animal as compared to averaging all individual traces from all individual behavioral events. This method allows each individual animal’s data to be weighed equally in the final quantification. (A) PETH shows increase in calcium signal in the dmPFC-DMS projection and decrease in calcium signal in the dmPFC-BLA projection during avoidance onset on Day 5. Dark grey line, mean ± SEM for dmPFC-DMS projection; light grey line, mean ± SEM for dmPFC-BLA projection; Grey box, baseline period (BL); yellow box, pre avoidance period (Pre); teal box, avoidance period (Avoid); pink box, post avoidance period (Post). (B) Quantification of avoidance PETH shows a significant increase in calcium signal in the avoid (0 to 3 s) period compared to baseline period (-10 to -7 s) for dmPFC-DMS projection. The dmPFC-BLA projection shows a significance decrease in signal during the avoid (0 to 3 s) and post avoid (7 to 10 s) periods compared to the pre-avoid period (-3 to 0 s) (Two-way ANOVA, Task Period x Projection F_(3, 60)_ = 7.528, p = 0.0002, Task Period p < 0.0001, Projection p < 0.0001; Sidak’s Multiple Comparisons Test, dmPFC-DMS Baseline vs dmPFC-DMS Avoid p = 0.0003, dmPFC-BLA Pre Avoid vs dmPFC-BLA Avoid p = 0.0439, dmPFC-BLA Pre Avoid vs dmPFC-BLA Post Avoid p = 0.0005, dmPFC-DMS Baseline vs dmPFC-BLA Baseline p > 0.9999, dmPFC-DMS Pre Avoid vs dmPFC-BLA Pre Avoid p = 0.9443, dmPFC-DMS Avoid vs dmPFC-BLA Avoid p < 0.0001, dmPFC-DMS Post Avoid vs dmPFC-BLA Post Avoid p = 0.0025, dmPFC-DMS N = 8 mice, n = 40 (8 mice x 5 days), dmPFC-BLA N = 9 mice, n = 45 (9 mice x 5 days)). (C) PETH shows no change in calcium signal at freezing onset for either the dmPFC-DMS or the dmPFC-BLA projection on Day 1. Dark grey line, mean ± SEM for dmPFC-DMS projection; light grey line, mean ± SEM for dmPFC-BLA projection; Grey box, baseline period (BL); Purple box, freezing period (Freezing). (D) Quantification of freezing PETH shows no significant change in calcium signal during the freezing period (0-0.5 s) compared to the baseline period (-2 to -1.5 s) (Two-way ANOVA, Task Period x Projection F_(1, 30)_ = 0.05096, p = 0.8229, Task Period p = 0.7429, Projection p = 0.0309; Sidak’s Multiple Comparisons Test, dmPFC-DMS Baseline vs dmPFC-BLA Baseline p = 0.6482, dmPFC-DMS Baseline vs dmPFC-DMS Freezing p > 0.9999, dmPFC-BLA Baseline vs dmPFC-BLA Freezing p = 0.6999, dmPFC-DMS Freezing vs dmPFC-BLA Freezing 0.4264; dmPFC-DMS N = 8 mice, n = 40 (8 mice x 5 days), dmPFC-BLA N = 9 mice, n = 45 (9 mice x 5 days)). ns = not significant, * p < 0.0332, ** p < 0.0021, *** p < 0.0002, **** p < 0.0001.


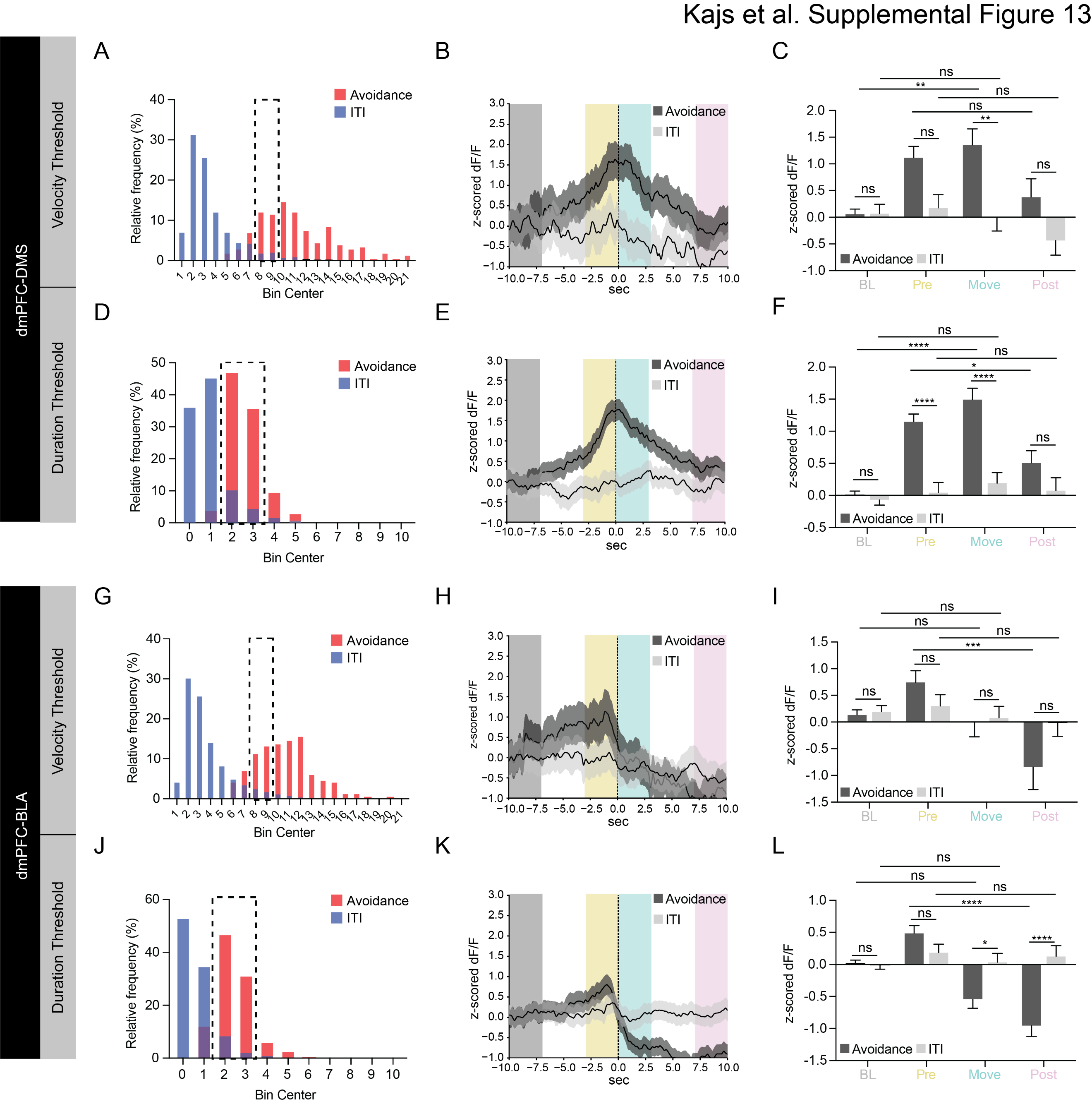


**Supplemental Figure 13.** Activity at avoidance onset in the dmPFC-DMS and dmPFC-BLA projections is not purely movement-related. (A) Distribution of movement velocities for ITI (blue) and avoidance (red) movements and their overlap (purple) for the dmPFC-DMS cohort. (B) PETH of ITI and avoidance movements of similar velocities (7.5 cm/s to 9.5 cm/s) aligned to movement onset shows increase in calcium signal during avoidance movements that is not seen during ITI movements in the dmPFC-DMS projection. Grey box, baseline period (BL); yellow box, pre movement period (Pre); teal box, movement period (Move); pink box, post movement period (Post) (C) Quantification of similar velocity movement PETH shows dmPFC-DMS calcium signal is significantly increased during avoidance movements compared to ITI movements during the movement (0 to 3 s) period, but not during baseline (-10 to -7 s), pre-movement (-3 to 0 s), or post-movement (7 to 10 s) periods (Two-way ANOVA, Task Period x Movement Type F_(3,332)_ = 2.620 p =0.0508, Task Period p = 0.0045, Movement Type p < 0.0001; Sidak’s Multiple Comparisons Test, Avoidance Baseline vs ITI Baseline p > 0.9999, Avoidance Baseline vs Avoidance Movement p = 0.0038, ITI Baseline vs ITI Movement p > 0.9999, Avoidance Pre-Movement vs ITI Pre-Movement p = 0.2121, Pre-Movement Avoidance vs Post-Movement Avoidance p = 0.5574, Pre-Movement ITI vs Post Movement ITI p = 0.9450, Avoidance Movement vs ITI Movement p = 0.0041, Avoidance Post-Movement vs ITI Post-Movement p = 0.4530; N = 8 mice, Avoidance n = 47 trials, ITI n = 38 trials). (D) Distribution of movement durations for ITI (blue) and avoidance (red) movements and their overlap (purple) for the dmPFC-DMS cohort. (E) PETH of ITI and avoidance movements of similar durations (1.5 s to 3.5 s) aligned to movement onset shows increase in calcium signal during avoidance movements that is not seen during ITI movements in the dmPFC-DMS projection. (F) Quantification of similar movement duration PETH shows dmPFC-DMS calcium signal is significantly increased during avoidance movements compared to ITI movements during the pre-movement (-3 to 0 s) and movement (0 to 3 s) periods, but not during the baseline (-10 to -7 s) or post-movement (7 to 10 s) periods (Two-way ANOVA, Task Period x Movement Type F_(3,1184)_ = 7.283, p < 0.0001, Task Period p < 0.0001, Movement Type p < 0.0001; Sidak’s Multiple Comparisons Test, Avoidance Baseline vs ITI Baseline p > 0.9999, Avoidance Baseline vs Avoidance Movement p < 0.0001, ITI Baseline vs ITI Movement p = 0.9993, Avoidance Pre-Movement vs ITI Pre-Movement p < 0.0001, Pre-Movement Avoidance vs Post-Movement Avoidance p = 0.0412, Pre-Movement ITI vs Post Movement ITI p > 0.9999, Avoidance Movement vs ITI Movement p < 0.0001, Avoidance Post-Movement vs ITI Post-Movement p =0.6948; N = 8 mice, Avoidance n = 162 trials, ITI n = 136 trials). (G) Distribution of movement velocities for ITI (blue) and avoidance (red) movements and their overlap (purple) for the dmPFC-BLA cohort. (H) PETH of ITI and avoidance movements of similar velocities (7.5 cm/s to 9.5 cm/s) aligned to movement onset shows decrease in calcium signal during avoidance movements that is not seen during ITI movements in the dmPFC-BLA projection. (I) Quantification of similar velocity movement PETH shows dmPFC-BLA calcium signal is significantly decreased during the post-movement (7 to 10 s) period compared to the pre-movement period (-3 to 0 s) for avoidance movements however there is no difference in dmPFC-BLA calcium signal during the baseline (-10 to -7 s), pre-movement (-3 to 0 s), movement (0 to 3 s), and post-movement (7 to 10 s) periods for ITI movements (Two-way ANOVA, Task Period x Movement Type F_(3,552)_ = 2.378, p = 0.0689, Task Period p = 0.0012, Movement Type p = 0.4383; Sidak’s Multiple Comparisons Test, Avoidance Baseline vs ITI Baseline p > 0.9999, Avoidance Baseline vs Avoidance Movement p > 0.9999, ITI Baseline vs ITI Movement p > 0.9999, Avoidance Pre-Movement vs ITI Pre-Movement p = 0.9975, Pre-Movement Avoidance vs Post-Movement Avoidance p = 0.0010, Pre-Movement ITI vs Post Movement ITI p > 0.9999, Avoidance Movement vs ITI Movement p > 0.9999, Avoidance Post-Movement vs ITI Post-Movement p = 0.3471; N = 9 mice, Avoidance n = 52 trials, ITI n = 88 trials) (J) Distribution of movement durations for ITI (blue) and avoidance (red) movements and their overlap (purple) for the dmPFC-BLA cohort. (K) PETH of ITI and avoidance movements of similar durations (1.5 s to 3.5 s) aligned to movement onset shows decrease in calcium signal during avoidance movements that is not seen during ITI movements in the dmPFC-BLA projection. (L) Quantification of similar movement duration PETH shows dmPFC-BLA calcium signal is significantly decreased during avoidance movements compared to ITI movements during the movement (0 to 3 s) and post-movement (7 to 10 s) periods, but not during the baseline (-10 to -7 s) or pre-movement (-3 to 0 s) periods (Two-way ANOVA, Task Period x Movement Type F_(3, 1496)_ = 11.80, p < 0.0001, Task Period p < 0.0001, Movement Type p = 0.0002; Sidak’s Multiple Comparisons Test, Avoidance Baseline vs ITI Baseline p > 0.9999, Avoidance Baseline vs Avoidance Movement p = 0.0815, ITI Baseline vs ITI Movement p > 0.9999, Avoidance Pre-Movement vs ITI Pre-Movement p = 0.9435, Pre-Movement Avoidance vs Post-Movement Avoidance p < 0.0001, Pre-Movement ITI vs Post Movement ITI p > 0.9999, Avoidance Movement vs ITI Movement p = 0.6149, Avoidance Post-Movement vs ITI Post-Movement p < 0.0001; N = 9 mice, Avoidance n = 165 trials, ITI n = 211 trials). ns = not significant, * p < 0.0332, ** p < 0.0021, *** p < 0.0002, **** p < 0.0001.
